# Divergent evolutionary processes associated with colonization of offshore islands

**DOI:** 10.1111/mec.12462

**Published:** 2013-09-03

**Authors:** Natália Martínková, Ross Barnett, Thomas Cucchi, Rahel Struchen, Marine Pascal, Michel Pascal, Martin C. Fischer, Thomas Higham, Selina Brace, Simon Y. W. Ho, Jean‐Pierre Quéré, Paul O'Higgins, Laurent Excoffier, Gerald Heckel, A. Rus Hoelzel, Keith M. Dobney, Jeremy B. Searle

**Affiliations:** ^1^ Department of BiologyUniversity of YorkWentworth WayYorkYO10 5DDUK; ^2^ Institute of Vertebrate BiologyAcademy of Sciences of the Czech RepublicKvětná 8Brno603 65Czech Republic; ^3^ School of Biological and Biomedical SciencesDurham UniversitySouth RoadDurhamDH1 3LEUK; ^4^ Department of ArchaeologyDurham UniversitySouth RoadDurhamDH1 3LEUK; ^5^ Department of ArchaeologyUniversity of AberdeenSt Mary's, Elphinstone RoadAberdeenAB24 3UFUK; ^6^ Muséum national d'histoire naturellecase postale 56 (bâtiment d'anatomie comparée)55 rue BuffonF‐75231 Paris Cedex 05ParisFrance; ^7^ Computational and Molecular Population Genetics (CMPG)Institute of Ecology and EvolutionUniversity of BernBaltzerstrasse 6BernCH‐3012Switzerland; ^8^ Équipe Écologie des Invasions BiologiquesINRACampus de Beaulieu 35 000Rennes CedexFrance; ^9^ Swiss Institute of BioinformaticsGenopodeLausanneCH‐1015Switzerland; ^10^ Oxford Radiocarbon Accelerator UnitResearch Laboratory for Archaeology and the History of Art (RLAHA)Dyson Perrins BuildingSouth Parks RoadOxfordOX1 3QYUK; ^11^ School of Biological SciencesRoyal HollowayUniversity of LondonEghamTW20 0EXUK; ^12^ School of Biological SciencesUniversity of SydneySydneyNSW2006Australia; ^13^ UMR CBGP (INRA/IRD/Cirad/Montpellier SupAgro)INRACampus international de BaillarguetCS 30016F‐34988 Montferrier‐sur‐Lez cedexFrance; ^14^ Centre for Anatomical and Human SciencesHull York Medical School (HYMS)University of YorkJohn Hughlings Jackson BuildingYorkYO10 5DDUK; ^15^ Department of Ecology and Evolutionary BiologyCorson HallCornell UniversityIthacaNY14853‐2701USA

**Keywords:** demographic analysis, genetic replacement, island colonization, *Microtus arvalis*, phylogeography

## Abstract

Oceanic islands have been a test ground for evolutionary theory, but here, we focus on the possibilities for evolutionary study created by offshore islands. These can be colonized through various means and by a wide range of species, including those with low dispersal capabilities. We use morphology, modern and ancient sequences of cytochrome *b* (*cytb*) and microsatellite genotypes to examine colonization history and evolutionary change associated with occupation of the Orkney archipelago by the common vole (*Microtus arvalis*), a species found in continental Europe but not in Britain. Among possible colonization scenarios, our results are most consistent with human introduction at least 5100 bp (confirmed by radiocarbon dating). We used approximate Bayesian computation of population history to infer the coast of Belgium as the possible source and estimated the evolutionary timescale using a Bayesian coalescent approach. We showed substantial morphological divergence of the island populations, including a size increase presumably driven by selection and reduced microsatellite variation likely reflecting founder events and genetic drift. More surprisingly, our results suggest that a recent and widespread *cytb* replacement event in the continental source area purged *cytb* variation there, whereas the ancestral diversity is largely retained in the colonized islands as a genetic ‘ark’. The replacement event in the continental *M. arvalis* was probably triggered by anthropogenic causes (land‐use change). Our studies illustrate that small offshore islands can act as field laboratories for studying various evolutionary processes over relatively short timescales, informing about the mainland source area as well as the island.

## Introduction

Marine islands were pivotal settings for the development of evolutionary theory (Wallace 1880) and continue to inspire evolutionary biologists today (Grant 2008). They tend to be ecologically simple and distinctive compared with mainland settings, with fewer competitors and predators, a different food supply and environmental conditions and a smaller living space, all factors that promote rapid and perhaps substantial evolutionary change relative to mainland source populations. Studies on islands have therefore provided insights into evolutionary processes such as species radiations (Emerson 2002) and the generation of evolutionary novelty (Lachaise *et al*. 2000).

Marine islands are also interesting for the varied ways in which they can be colonized. First, for islands separating from a mainland, some species may already be present at island formation. Second, islands may be colonized by natural ‘sweepstake’ dispersal (Simpson 1940). Finally, species may be introduced into islands by humans.

Many of the classic systems for the study of island evolution, particularly species radiations, have involved oceanic islands or archipelagos colonized by sweepstake dispersal, for example Darwin's finches on the Galapagos and Hawaiian drosophila. However, here we consider offshore islands, which can be colonized by all three mechanisms, providing rich opportunities for evolutionary studies, notably of poor dispersers such as small mammals (Berry 1996).

Offshore islands around the Atlantic coast of Europe were formed by end‐glacial sea level rise, and thus, small mammals currently found on them may have already been present prior to island separation, and perhaps even before the last glacial maximum (LGM; *c*. 20 000 bp: Ehlers & Gibbard 2004), if local conditions were compatible with survival (Beirne 1952; Martínková *et al*. 2007). Alternatively, the small mammals could have been introduced by humans, for example, within bedding/fodder for livestock (Corbet 1961). Sweepstake dispersal, by rafting on floating masses of vegetation (Morrison *et al*. 1992) or walking over ice (White & Searle 2008), is also a possibility.

**Figure 1:**
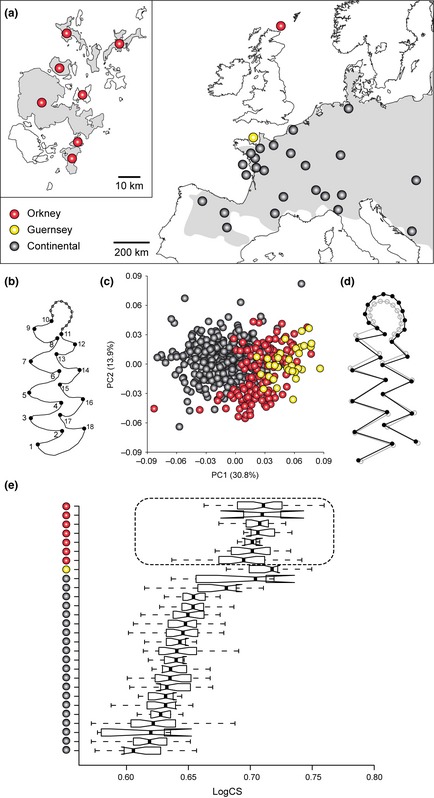
The morphological variation of the first lower molar (*M*_1_) among modern *Microtus arvalis* populations. (a) Sampling localities within its western and central European range (Shenbrot & Krasnov 2005) (in grey). Note that the three Spanish localities and the three Italian localities are treated as single entries in Fig. 1e and Table S1 (Supporting information). (b) Position of the 18 landmarks and 12 semi‐landmarks on the *M*_1_ occlusal view. (c) Scatter plot of the first two principal component scores depicting overall *M*_1_ shape variation. (d) Diagram showing the *M*_1_ shape change associated with the PC1 scores by 0.1 units in the positive direction (black) against the mean shape (grey). (e) Box plot showing log‐transformed centroid size variation of the *M*_1_.

In this study, we aim to distinguish between these different modes of colonization of a European offshore archipelago (Orkney) by a small mammal (the common vole *Microtus arvalis*) and to document evolutionary change in that species postcolonization. *Microtus arvalis* is a ground‐living, grass‐eating small rodent that is common in agricultural meadows (Niethammer & Krapp 1982). The species is widespread in western continental Europe, although it is absent from Britain and Fennoscandia (Fig. 1a). Therefore, the whole British landmass separates the *M. arvalis* populations in continental Europe and on Orkney. The only species of *Microtus* in mainland Britain and other islands off mainland Britain is *Microtus agrestis*, which, like *M. arvalis*, has a wide continental European range (Shenbrot & Krasnov 2005). The two species belong to unrelated lineages within *Microtus* (Fink *et al*. 2010; Martínková & Moravec 2012).

The colonization history of a population can be inferred through comparisons with potential source populations. If the presence of the Orkney vole is explained by glacial survival or sweepstake dispersal, then surviving representatives of the source populations are no longer available, because there are no extant populations suitably close to Orkney. However, human introduction can come from a distance, and the most likely source area is represented by the closest coastal part of the continental European range of the same mitochondrial lineage as found on Orkney, that is, northern France and Belgium (Haynes *et al*. 2003; Tougard *et al*. 2008). For this reason, we focus on this potential source area in our analyses and consider whether such a human introduction is feasible or whether one of the other modes of colonization is more likely.

To explore the colonization history of the Orkney vole and the evolutionary change since colonization, we compare populations from Orkney and the potential source area using mitochondrial DNA sequences and microsatellites. We also use archaeological deposits of the species from Orkney for ancient DNA (aDNA) sequencing and radiocarbon dating (two Orkney vole bones have previously been radiocarbon‐dated [Hedges *et al*. 1987] using technology now outdated), to obtain a more detailed temporal dimension to the colonization history and to support a demographic analysis. Finally, we use geometric morphometrics of a molar tooth as a marker for morphological evolution. This work provides a more sophisticated assessment of colonization and evolution of the Orkney vole than has previously been possible (Berry & Rose 1975; Corbet 1986; Haynes *et al*. 2003; Thaw *et al*. 2004; Gorman & Reynolds 2008). More generally, this case study provides new insights into not only short‐term evolutionary changes in offshore islands, but also the mainland areas with which they are being compared.

## Materials and methods

### Specimens

**Figure 2:**
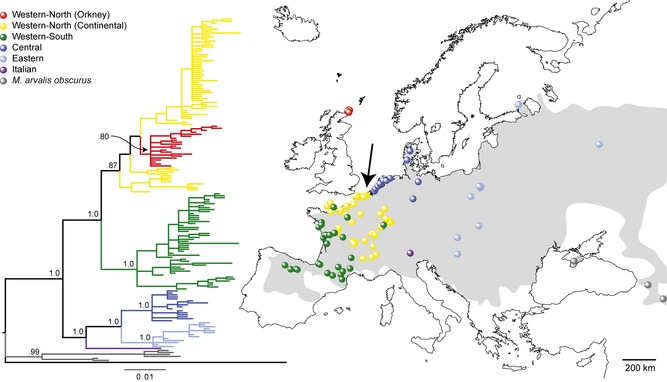
Bayesian phylogenetic tree depicting all complete cytochrome *b* haplotypes of modern *Microtus arvalis* and localities where each phylogroup is found. Lineages labelled following previous authors (Haynes *et al*. 2003; Tougard *et al*. 2008): Orkney (red) within Western‐North (yellow), Western‐South (green), Central (dark blue), Eastern (light blue), Italian (purple), *M. a. obscurus* (grey). Posterior probability is presented for major clades only. Outgroup sequence in tree: *Microtus levis*, AY513819 (Jaarola *et al*. 2004). Grey shading: distribution of *M. arvalis* (Shenbrot & Krasnov 2005) where three *obscurus* localities are off the map to the east. Black arrow shows the most likely source locality of the Orkney voles, based on microsatellite data (see Table 1).

Details of all *Microtus arvalis* specimens used in this study are listed in Tables 1 and 2 and Tables S1–S3 (Supporting information) and mapped in Figs 1 and 2 and Figs S1 and S2 (Supporting information). Geometric morphometrics were applied to 553 modern specimens from 27 localities, while 651 *M. arvalis* specimens from 70 localities were used for the analysis of modern DNA (125 specimens were used in both *cytb* and microsatellite studies). Ancient DNA analysis was conducted on 37 archaeological specimens from eight localities. Nineteen of these were among 23 archaeological specimens used for radiocarbon dating, calibrated using Calib Rev. 5.0.1 (Stuiver & Reimer 1993) and IntCal04 (Reimer *et al*. 2004), with dates before present (bp) standardized to 1950 AD.

**Table 1:** Microsatellite comparison of *Microtus arvalis* in Orkney and continental Europe, including diversity indices for all populations sampled and approximate Bayesian computation selection of most likely continental population for the Orkney colonization. For map of localities, see Fig. S1 (Supporting information)

Locality	Country/Orkney Island	Latitude	Longitude	Number of specimens	mtDNA lineage	A_R_	H	∆_*C*_
Stalhille	Belgium	51.21	3.07	26	Western‐North	5.69 (1.90)	0.72 (0.15)	2.03
Pihen lès Guînes	France	50.87	1.79	22	Western‐North	7.02 (2.08)	0.78 (0.17)	2.24
Daubeuf	France	49.78	0.52	20	Western‐North	6.65 (2.51)	0.73 (0.25)	2.24
Alflen	Germany	50.18	7.04	22	Western‐North	6.62 (1.66)	0.80 (0.09)	2.27
Clérmont‐Ferrand	France	45.78	3.08	23	Western‐North	6.06 (2.21)	0.70 (0.23)	2.62
Avallon	France	47.85	3.90	22	Western‐North	6.74 (1.84)	0.78 (0.13)	2.63
Aiffres	France	46.29	−0.41	22	Western‐South	7.59 (3.34)	0.73 (0.27)	2.66
Fressenneville	France	50.07	1.58	24	Western‐North	6.84 (2.23)	0.73 (0.23)	2.71
Thaon	France	49.26	−0.46	24	Western‐North	7.19 (2.76)	0.74 (0.24)	2.72
Ste Marie du Mont	France	49.38	−1.23	20	Western‐North	6.72 (2.88)	0.69 (0.28)	2.91
Schiltach	Germany	48.30	8.34	20	Western‐North	5.30 (1.58)	0.74 (0.11)	2.94
Veurne	Belgium	51.07	2.66	26	Western‐North	4.09 (0.95)	0.65 (0.11)	3.02
Baie d'Aiguillon	France	46.30	1.17	15	Western‐South	7.76 (3.38)	0.74 (0.26)	—
St Jean le Thomas	France	48.73	−1.51	10	Western‐North	4.07 (2.13)	0.59 (0.24)	—
Dinteloord	Netherlands	51.64	4.37	12	Central	4.68 (1.59)	0.66 (0.24)	—
Heerenveen	Netherlands	52.96	5.93	13	Central	6.04 (2.08)	0.75 (0.23)	—
Harray Stenness	Mainland, Orkney	59.02	−3.20	23	Western‐North	5.01 (2.31)	0.62 (0.26)	—
Settiscarth	Mainland, Orkney	59.05	−3.10	26	Western‐North	4.35 (1.88)	0.60 (0.27)	—
St Ola	Mainland, Orkney	58.94	−2.95	19	Western‐North	3.79 (1.94)	0.50 (0.31)	—
Whitemill Bay	Sanday, Orkney	59.30	−2.55	19	Western‐North	2.11 (1.60)	0.23 (0.26)	—
Wind Wick	S Ronaldsay, Orkney	58.76	−2.94	20	Western‐North	1.83 (1.14)	0.21 (0.26)	—
Grimness	S Ronaldsay, Orkney	58.82	−2.91	20	Western‐North	2.17 (1.21)	0.24 (0.26)	—
Loch of Swartmill	Westray, Orkney	59.29	−2.92	21	Western‐North	2.16 (1.50)	0.26 (0.31)	—
Ness	Westray, Orkney	59.24	−2.87	23	Western‐North	1.73 (1.19)	0.19 (0.29)	—

**Figure 3:**
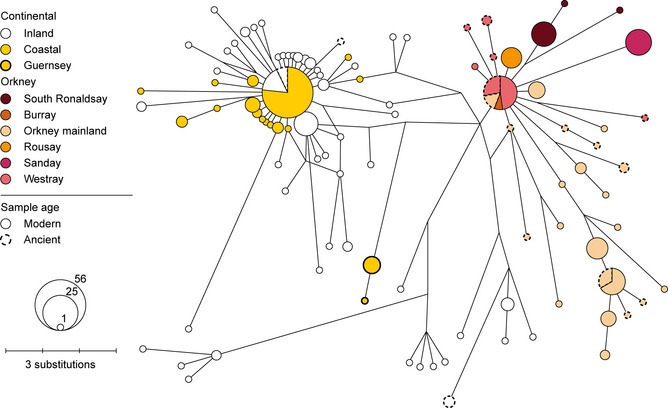
Median‐joining network showing all modern and ancient DNA haplotypes in the Western‐North phylogroup of *Microtus arvalis*. The phylogroup is defined in Fig. 2. Node size is proportional to haplotype frequency and edge length to number of substitutions separating the haplotypes. Guernsey is an offshore island also analysed in the geometric morphometric analysis (see Fig. 1).

A_R_: mean allelic richness over loci. H: mean heterozygosity over loci; values in parentheses are standard deviations. Δ_*C*_: average distance between the observed and 1000 simulated summary statistics computed over the three Mainland Orkney populations for each continental population (c) to find the most likely source population for the colonization of Orkney by *M. arvalis* (smallest value). Only samples of 19 or more individuals were used for this analysis. The populations on Mainland Orkney were considered to best represent the colonized area, because of retention of high diversity (see also Fig. 3).

**Table 2:** ^14^C dates and their calibrated age ranges for 23 *Microtus arvalis* mandibles collected from Orkney

Laboratory number	Sample reference	Site name	Island	^14^C age bp	95.4% (2s) cal age ranges
OxA 18324	R44	Point of Cott	Westray	4555 ± 40	cal bp 5050–5437
OxA 18782	R37	Point of Cott	Westray	4459 ± 33	cal bp 4967–5288
OxA 18325	R45	Point of Cott	Westray	4451 ± 38	cal bp 4884–5287
OxA‐18668	R177	Quanterness	Mainland	4414 ± 27	cal bp 4869–5257
OxA‐18784	R179	Quanterness	Mainland	4400 ± 33	cal bp 4861–5213
OxA‐18786	R191	Skara Brae	Mainland	4199 ± 33	cal bp 4622–4844
OxA‐20309	R84	Skara Brae	Mainland	4145 ± 29	cal bp 4574–4823
OxA‐18664	R11	Skara Brae	Mainland	4124 ± 28	cal bp 4529–4815
OxA‐18663	R3	Skara Brae	Mainland	3946 ± 27	cal bp 4294–4515
OxA‐18787	R194	Skara Brae	Mainland	3939 ± 32	cal bp 4256–4514
OxA‐18669	R193	Skara Brae	Mainland	3906 ± 27	cal bp 4249–4419
OxA‐18785	R189	Skara Brae	Mainland	3884 ± 31	cal bp 4185–4418
OxA‐18666	R23	Holm of Papa Westray	Westray	4089 ± 29	cal bp 4448–4808
OxA‐18665	R20	Holm of Papa Westray	Westray	4054 ± 28	cal bp 4435–4784
OxA 18328	R126	Pierowall Quarry	Westray	4000 ± 45	cal bp 4298–4781
OxA 18783	R124	Pierowall Quarry	Westray	3824 ± 34	cal bp 4094–4406
OxA 18327	R99	Pierowall Quarry	Westray	3822 ± 38	cal bp 4092–4406
OxA‐18350	R58	Howe	Mainland	1860 ± 28	cal bp 1721–1869
OxA‐18351	R59	Howe	Mainland	1849 ± 27	cal bp 1714–1865
OxA‐18667	R62	Howe	Mainland	1469 ± 24	cal bp 1308–1396
OxA‐20310	RH3	Green Hill, South Walls	Hoy	1100 ± 24	cal bp 958–1060
OxA‐20481	RH2	Green Hill, South Walls	Hoy	993 ± 27	cal bp 798–962
OxA 18326	R29	Earl's Bu	Mainland	966 ± 29	cal bp 795–932

### Geometric morphometrics

Modern *M. arvalis* from populations in Orkney and continental Europe (Fig. 1a) were compared by geometric morphometrics of dental phenotype. Digital photographs of the first lower molar (*M*_1_) were used to record two‐dimensional Cartesian coordinates of 18 anatomical landmarks at the bases and tips of the lingual and labial cusps as well as 12 equidistant semi‐landmarks on a manually drawn curve along the anterior loop (Fig. 1b).

Digitization of landmarks and semi‐landmarks was performed using TPSdig 2 (Rohlf 2010a). Position, orientation and scaling information from the raw coordinates were standardized by a generalized Procrustes analysis (GPA) using TPSrelw 1.49 (Rohlf 2010b). To combine landmarks and semi‐landmarks in the GPA, the semi‐landmarks along the anterior loop curve were constrained to slide along an estimated tangent at each sliding point using the bending energy method (Bookstein 1997).

The overall size parameter for the *M*_1_ is centroid size (square root of the sum of squared distances of landmarks and semi‐landmarks from the centroid) and comparisons between populations use a box plot of log‐transformed values produced with ‘R’ v. 2.13.1 (R Development Core Team 2011). The shape variables are the Procrustes coordinates obtained after the GPA, and variation among populations is displayed by principal component analysis (PCA) using MorphoJ 1.05c (Klingenberg 2011).

### Cytochrome *b* sequencing (modern DNA)

A total of 283 specimens of *M. arvalis* were sequenced from 18 localities in Orkney and 50 localities in continental Europe (particularly from around the potential area of human introduction: northern France and Belgium and inland from there). Total genomic DNA was isolated using the DNeasy Tissue Kit (Qiagen, Halden, Germany) and PCR‐amplified using previously described primers (Table S4, Supporting information; Jaarola *et al*. 2004). PCR products were purified with QIAquick PCR purification kit (Qiagen) and commercially sequenced using BigDye Terminator Sequencing chemistry (Applied Biosystems, Foster City, CA, USA) with newly designed primers (Table S4, Supporting information) and run on ABI PRISM 3730xl sequencers (Applied Biosystems). Complete *cytb* sequences (1143 bp) were generated (GU190383–GU190665).

### Cytochrome *b* sequencing (ancient DNA)

All aDNA extractions were performed in a laboratory in Durham where no modern molecular biology or post‐PCR work is undertaken and where *Microtus* were analysed for the first time. Before use, all materials and work surfaces were wiped with 10% bleach, and the workspace was UV‐irradiated overnight. Samples of *Microtus* bone were excised using a scalpel blade and crushed within aluminium foil. The bone powder was incubated overnight on a rotator at 55 °C in 500 μL of extraction buffer (0.5m pH 8.0 EDTA, 0.1m pH 8.0 Tris, 0.05% w/v SDS) with 8 μL of proteinase K (0.3 mg/mL). Digested samples were then extracted using the Qiagen QIAquick PCR purification method, as described in Nichols *et al*. (2007). Final elutions of aDNA were collected in 50 μL of TE buffer following the QIAquick protocol and stored at −20 °C. Negative extraction controls (lacking bone powder) were also performed in parallel in a ratio of approximately 1:10.

Ancient DNA was successfully obtained from 37 specimens (of 190 attempted) from Neolithic to Viking Age Orkney and Late Medieval Belgium (Table S3, Fig. S2, Supporting information). Partial *cytb* sequences (1130 bp) were generated through 8–9 overlapping PCRs (Table S4, Supporting information), with the missing *cytb* sequence at the 5′‐end. Each PCR used 2 μL of aDNA extract in a 25 μL volume with Hi‐Fidelity Platinum *Taq* (Invitrogen, Carlsbad, CA, USA). Purification and sequencing of PCR products followed the protocols used for modern DNA.

As further checks on the aDNA authenticity additional to the negative extraction and PCR controls, two samples were internally replicated from bone powder (R59 and R60), producing identical sequences. All sequences with unique mutations not found in modern Orkney voles were amplified at least twice for confirmation. The regions of overlap between the PCR products were checked and found to be consistent. The assembled *cytb* coding regions for all ancient voles produced a readable protein sequence with no frameshift or nonsense mutations. *M. arvalis* is known to possess a nuclear copy of *cytb* (DeWoody *et al*. 1999) that is easily identifiable due to unique fixed mutations and insertions and was not amplified from any of the aDNA extracts.

Six samples (R44, R58, R125, R131, R189 and R191) were sent to Egham where the entire procedure, including extraction and amplification, was replicated. Two samples (R125 and R131) were sequenced in their entirety and produced identical sequences to those in Durham. Radiocarbon dating and high collagen levels found in 19 samples from Orkney that had been successful for aDNA extraction give corroborative evidence for good biochemical preservation of the samples.

### Microsatellites

A total of 492 modern specimens from eight localities in Orkney and 16 localities in continental Europe from around the most probable area of introduction (northern France and Belgium and inland from there; Fig. S1, Supporting information) were typed at 14 nuclear microsatellite loci (Heckel *et al*. 2005; Jaarola *et al*. 2007; Walser & Heckel 2008): CRB5, INb, MAG6, MAG25, MAR003, MAR016, MAR063, MAR076, MAR080, MAR102, MAR113, MM1, MM8 and Moe02. PCR amplifications were performed with the Qiagen Multiplex kit using Gene Amp PCR System 9700 (Applied Biosystems), PCR fragments were separated on an ABI 3100 sequencer (Applied Biosystems), and their lengths determined using genemapper 3.7 (Applied Biosystems).

For each locus and each population, tests for departure from Hardy–Weinberg equilibrium were performed with arlequin 3.12 (Excoffier *et al*. 2005), and significance levels were corrected for multiple testing using the sequential Bonferroni–Holm procedure (Rice 1989). There were no significant departures from equilibrium. Mean allelic richness over loci (A_R_) was calculated with fstat version 2.9.3.2 (Goudet 1995) and the mean heterozygosity (H) over loci and population differentiation (*F*_ST_) with Arlequin.

### Phylogenetic analysis

For *cytb*, sequence chromatograms were assembled in Sequencher 4.5 (GeneCodes, Ann Arbor, MI, USA) and aligned manually in BioEdit (Hall 1999). Newly obtained modern DNA sequences were analysed together with previously published modern sequences (Martin *et al*. 2000; Haynes *et al*. 2003; Borkowska & Ratkiewicz 2008; Tougard *et al*. 2008). Using the 1143‐bp alignment, this yielded a total of 395 sequences distributed among 173 haplotypes. These were derived from 124 localities (106 from continental Europe, 18 from Orkney). *Microtus levis* was used as an outgroup (Martínková & Moravec 2012).

A Bayesian phylogenetic tree of all modern sequences with the complete (1143 bp) *cytb* was estimated in MrBayes 3.1.2 (Ronquist & Huelsenbeck 2003). To avoid long‐tree artefact (Marshall 2010), the Γ‐distribution shape parameter α was fixed to 1.0736. The value was obtained from the GTR+Γ substitution model selected by Akaike information criterion in MrModeltest 2.3 (Posada & Crandall 1998; Nylander 2004). The two separate Markov chain Monte Carlo (MCMC) analyses each comprised one cold and eleven heated chains. Samples were drawn from the posterior every 1000 steps over 10 million steps. The chain temperature was 0.06, and two chain swaps were attempted every step to optimize mixing. The first 3000 sampled trees were discarded as burn‐in, with the resulting average standard deviation of split frequencies equal to 0.007. The 95% credibility interval of the tree length included a maximum‐likelihood estimate of the tree length obtained from raxml 7.2 (Stamatakis 2006).

Median‐joining networks were constructed in Network 4.2 (Bandelt *et al*. 1999) with an equal transition/transversion ratio. The analysis was carried out using the 1130‐bp alignment because aDNA sequences were included.

### Demographic analysis

For *cytb*, nucleotide and haplotype diversities (Nei 1987) and Ramos‐Onsins & Rozas's (2002) *R*_2_ were determined using dnasp 5.1 (Rozas *et al*. 2003). The neutrality test statistics Tajima's (1989) *D* and Fu's (1997) *F*_S_ were estimated in Arlequin. This software was also used for mismatch distribution analysis (Rogers & Harpending 1992) to detect and date population expansions.

Radiocarbon‐dated and modern *M. arvalis cytb* sequences were analysed using the program beast 1.5.1 (Drummond & Rambaut 2007), utilizing the 1130‐bp alignment because of inclusion of aDNA sequences. For the Orkney data set, the GTR+Γ model of nucleotide substitution was selected using the Akaike information criterion. All beast analyses used this model, along with a strict molecular clock. A comparison using Bayes factors selected the constant‐size coalescent prior as the most appropriate demographic model. With ancient sequences in the data set, the potential for undetected sequence errors to influence the analyses was also modelled in beast (Rambaut *et al*. 2009). All other parameters were co‐estimated with the phylogeny, with samples drawn from the posterior every 1000 steps over a total of 10 million steps. The first 1 000 000 steps were discarded as burn‐in. Acceptable mixing and convergence to stationarity were checked using the program tracer 1.4.1 (Rambaut & Drummond 2007).

For modern DNA sequences from continental European populations, we used a demographic model simulating population expansion and used Bayes factors to compare a strict molecular clock, uncorrelated lognormal relaxed clock and uncorrelated exponential relaxed clock (Drummond *et al*. 2006). A model of exponential growth was identified as the best‐fitting demographic model using Bayes factors. Samples were drawn from the Markov chain every 10 000 steps over a total of 100 million steps, with the first 30% of sampled trees discarded as burn‐in. Estimated effective sample sizes were >200. Mean mutation rate was fixed to that obtained from the tip‐dated sequence analysis described above, and each lineage that was expected to exhibit unique demographic history in the target time frame was analysed separately.

For the specific question of time of colonization of Orkney, we used the program IMa (Hey & Nielsen 2007) to estimate the splitting time between the modern Orkney sample (N* *=* *57 for mtDNA, N* *=* *114 for microsatellite loci) and European mainland samples from the potential area of human introduction: northern France, Belgium and nearby areas of Germany (N* *=* *46 for mtDNA, N* *=* *92 for microsatellite loci). Initial runs were performed to estimate parameters, followed by three replicate runs with different random number seeds to check for consistency of results (results from the final run reported). Input data were 1143‐bp *cytb* sequences (assuming the HKY mutation model and the mean mutation rate estimated in beast; priors on the range of mutation rate scalars were set one order of magnitude above and below) and 14 microsatellite loci. The inheritance scalar was set at 0.25. Metropolis coupling was implemented using 20 chains and a geometric heating model (term 1: 0.99; term 2: 0.95). The burn‐in period was 7 h. Upper bounds were set for prior distributions for theta, migration rate and the splitting time. The final run saved 499 469 trees per locus and ran for 705 h (50 million steps following burn‐in). Convergence was tested by ensuring that effective sample sizes exceeded 50 and parameter trend lines were flat.

We analysed the microsatellite data using approximate Bayesian computation (ABC) to estimate demographic parameters of the colonization history of Orkney by *M. arvalis*. Comparisons were made between the continental European and Mainland Orkney populations listed in Table 1 (and mapped in Fig. S1, Supporting information), except that the four continental populations with small sample sizes (15 individuals or fewer) were excluded. Again, we focused on populations from the region of continental Europe that most likely represented a source for the introduction. We specified an ABC model with 11 demographic parameters in which the Orkney population diverged from a continental population after a bottleneck. Moreover, both the Orkney and the continental populations were allowed to pass through independent bottlenecks. The bottleneck of the continental population could occur either before or after the colonization of Orkney. For simplicity, the model was simulated with instantaneous growth after all three bottleneck events. Further details are given in Data S1, Table S5 and Fig. S3 (Supporting information).

## Results

### Characteristics of the Orkney voles

Among extensive samples of *Microtus arvalis* collected from across their European range, it is clear that the island populations on Orkney (seven samples) and Guernsey (one sample) are highly distinctive compared with their continental counterparts in first lower molar morphology (Fig. 1). This relates to both size (the island voles generally had larger teeth: Fig. 1e) and shape (the Orkney voles were divergent from continental and insular European *M. arvalis* in the principal components scatter plot, with the *M*_1_ displaying a relatively broader anterior loop: Fig. 1c, d).

At the 14 microsatellite loci screened, the mean number of alleles per locus and heterozygosities were systematically lower in the Orkney populations than in northern France, Belgium and nearby areas of Germany (Table 1), the areas of continental Europe from where introduced voles most likely originated. There was no overlap in heterozygosity values and the small overlap in mean number of alleles per locus related to Mainland Orkney populations, which had distinctly higher diversity values than other Orkney Island populations.

Overall *F*_ST_ between population samples was very high (0.382, *P *<* *0.0001) in agreement with previous studies on the species (e.g. Heckel *et al*. 2005). Pairwise comparisons between Orkney populations ranged from *F*_ST_ = 0.094 to 0.682 (mean: 0.412) and between 0.007 and 0.346 for the continental populations (mean: 0.177; Table S6, Supporting information).

### Special features of the mitochondrial DNA variation

Together with previously published results, our new data confirm that *M. arvalis* of the same (Western‐North) *cytb* lineage found today in France, Belgium, the Netherlands, Germany and Switzerland also occurs on Orkney (Fig. 2 and Table S2, Supporting information). Fig. 2 shows very clearly that the coastline of France and Belgium provides possible maritime access of the Western‐North *cytb* lineage to Orkney, consistent with our contention that this is the most likely source region for an introduction of *M. arvalis* there.

In the Bayesian phylogenetic tree (Fig. 2) and the median‐joining network (Fig. 3), the Orkney *cytb* sequences form an unsupported monophyletic clade within the Western‐North lineage. However, within that context, the sequences are very distinct from those from coastal Belgium/France and other regions in continental Europe, separated by at least four mutations. Thus, despite detailed sampling of the Western‐North lineage in general and along the coast of France and Belgium in particular (Fig. 2), we found no *cytb* sequences there that are clearly ancestral to those of the Orkney voles.

Instead, over this coastal region, an area of more than 10 000 km^2^ (Table 3), a single mtDNA haplotype (and sequences separated by one mutation from it) is dominant. None of these haplotypes relate closely to the Orkney *M. arvalis* haplotypes (Fig. 3). This ‘starburst’ of the coastal French and Belgian sequences suggests their recent expansion and dispersal. A significant deviation from neutral expectation for Tajima's *D* and Fu's *F*s, a small Rozas's *R*_2_ and a significant signal in pairwise DNA sequence comparisons in the mismatch distribution are consistent with this (Table 3).

**Table 3:** Modern mtDNA comparison of *Microtus arvalis* in Orkney and continental Europe

	Coastal France and Belgium sampled^a^	Orkney Mainland	All Orkney Islands sampled
Total area (km^2^)	10 439	52	73
Number of individuals sequenced	68	42	97
Haplotype diversity (*h * ± SD)	0.785 ± 0.039	0.864 ± 0.029	0.911 ± 0.108
Nucleotide diversity (π ± SD)	0.00127 ± 0.00017	0.00356 ± 0.00201	0.00416 ± 0.00227
Tajima's *D*	**−1.986**	**−**1.102	**−**1.167
Fu's *F*_S_	**−10.035**	**−**1.455	**−**3.014
Rozas's *R*_2_	0.1616	0.0739	0.0587
Mismatch distribution (SSD)	**0.0042**	**0.0112**	**0.0037**

Characteristics of the modern mtDNA sequences of *M. arvalis* from the Orkney archipelago and coastal regions of France and Belgium (where the Orkney *M. arvalis* most likely originated, see text) relating to the population expansion in the two areas.

For neutrality test statistics and the mismatch distribution, significant values (*P *<* *0.01) consistent with population expansion are given in bold.

a This included all localities within 100 km of the coast. The area sampled was estimated conservatively as a rectangle with one side given by the distance between the end localities along the coast and the other by the mean distance to the coast of all localities.

Considering now the Orkney sequences, the same *cytb* lineage and two of the same haplotypes as those in modern specimens were also found in *M. arvalis* dating back to the Neolithic period (Fig. 3; Table S3, Supporting information). Interestingly, the central Orkney haplotype found in archaeological specimens from Mainland Orkney and Westray was detected in modern specimens from Westray and Burray not Mainland Orkney. A second haplotype was found in both archaeological and modern sequences from Mainland Orkney. Both archaeological and modern sequences from Mainland Orkney are represented by many haplotypes in the network (9 and 12, respectively), and there are five archaeological and two modern haplotypes from Westray (Fig. 3).

### A possible source area for the introduction of *M. arvalis* to Orkney

We used ABC on the microsatellite data to compare 12 current populations (both coastal and inland) from northern France, Belgium and nearby areas of Germany as possible source populations for Orkney voles (Table 1 and Fig. S1, Supporting information). Our results confirmed that, if *M. arvalis* were introduced to Orkney from this source region, they most likely originated from coastal Belgium/France (three of the four best‐supported populations: Table 1) rather than inland continental populations. The best‐supported modern source population is from Stalhille on the Belgian coast (Fig. 2 and Table 1) and geographically the closest point to Orkney within the distribution of the Western‐North lineage.

### Time of colonization of Orkney

We obtained new ^14^C dates for 23 specimens from archaeological sites dating from the Late Neolithic period to the Viking Age (Table 2). The estimated dates are consistent with those of the archaeological contexts from which their bones were recovered. They show definitively that *M. arvalis* was present on Orkney at least 5100 bp. Two molecular analyses suggest that they did not arrive much earlier than this. An ABC analysis of microsatellites shows modal point estimates of time of colonization of Orkney that vary between 3000 and 5700 bp (between Stalhille, Belgian coast and three localities on Mainland Orkney). An IMa analysis (Hey & Nielsen 2007) of ancient and modern mtDNA sequences and microsatellites yields an estimated colonization time of 4515 years (90% CI: 2568–9019). Both analyses assume a single generation per year. Multiple generations (for which there is no evidence: Gorman & Reynolds 2008) would imply a more recent post‐Neolithic colonization, which does not fit well with our new ^14^C dates (voles on Orkney at latest 5100 bp) and aDNA sequencing of archaeological specimens (same genetic lineage as the modern specimens).

While the radiocarbon and molecular dating indicate that voles arrived on Orkney about 5000 bp, the time to the most recent common ancestor (tMRCA) for the Orkney *cytb* sequences is much earlier, *c*. 15 400 years (95% CI of 8100–25 400 years), based on Bayesian coalescent analysis incorporating modern and ancient DNA data. Using the same mutation rate (*μ *= 3.27 × 10^−7^ mutations/site/year) as for Orkney sequences, the tMRCA for the sequences from coastal France/Belgium (the putative source area for the Orkney voles) is only 2356 years (95% CI of 723–4941 years). Mismatch analysis (Rogers & Harpending 1992) provides further evidence of the recentness of the derivation of the *cytb* sequences in this potential source area for the Orkney voles, showing an expansion beginning 1860 bp (95% CI: 1201–2665).

## Discussion

### *Cytb* mutation rate

Estimates of mutation rates for phylogeographical studies have traditionally been based on fossil calibrations, often founded on very incomplete information. For *Microtus*, Triant and DeWoody (2006) used a fossil calibration point of 1.5 Ma to provide a mutation rate for *cytb* of approximately 8 × 10^−8^ mutations/site/year. Although this is probably the best‐substantiated fossil‐based estimate of the *cytb* mutation rate for *Microtus* in the literature, we considered it inadequate for our needs. This is because the fossil calibration point likely has errors (it is not based on precisely dated geological events or fossil transitions) and is geologically very much earlier than the time frame for our study. Instead, we were able to calibrate our rate estimate using the aDNA sequences from radiocarbon‐dated specimens as part of a Bayesian coalescent analysis. This method has the advantage of producing an estimate of the *cytb* mutation rate relevant to precisely the population that we were studying and the temporal scale of interest.

The mutation rate we obtained and used here (3.27 × 10^−7^ mutations/site/year) was about four times higher than that of Triant and DeWoody (2006), but comparable to other aDNA‐based estimates of mutation rate for a variety of species (Ho *et al*. 2011). Navascués & Emerson (2009) caution against possible upward bias of such aDNA‐based estimates under extreme demographic scenarios, but there is a growing consensus in the literature that aDNA‐based estimates are better than traditional fossil‐based mutation rates, which are too low for dating divergences and demographic events that have occurred over the last few thousand years (Ho & Larson 2006; de Bruyn *et al*. 2011). With regard to our aDNA‐based *cytb* mutation rate for *M. arvalis*, it is extremely close to that obtained for another *Microtus*, which used a very recent geological event as a calibration point (*M. agrestis*; 3.89 × 10^−7^ mutations/site/year; Herman & Searle 2011). This provides independent support for our aDNA‐based estimate, although, as with all molecular dating, interpretations have to be viewed with caution.

### Colonization history of Orkney voles

It is striking that there is substantial *cytb* variation in *M. arvalis* in Mainland Orkney and over the whole archipelago (Fig. 3, Table 3). Island populations often show low genetic diversity (Frankham 1997). This can relate to small population sizes and/or population bottlenecks associated with colonization of islands, particularly by sweepstake dispersal or human introduction. The high *cytb* diversity could indicate that the Orkney population of *M. arvalis* represents an island relict of a previously continuous mainland population, perhaps dating back to before the LGM (Beirne 1952). This would fit with the long tMRCA for the molecular variation on Orkney, potentially dating back to 25 400 bp (within the 95% CI).

However, there are strong arguments against glacial survival of the Orkney vole population. First, the IMa analysis based on microsatellites and *cytb* and the ABC analysis based on microsatellites provide a date of arrival around 5000 bp, considerably more recent than the LGM. Second, all the other species of small mammals on Orkney are most reasonably viewed as human introductions (Yalden 1982), necessitating a special case for *M. arvalis* as a glacial survivor. Third, it is very difficult to make this special case given that *M. arvalis* is not a species currently associated with arctic or even moderately high latitude conditions. Its range extends eastward beyond Lake Baikal and yet barely traverses north of the 60th parallel (Fig. 2; Shenbrot & Krasnov 2005). Orkney was under or near a glacial ice sheet at the LGM (Bowen *et al*. 2002) and *M. arvalis* is not part of the fossil fauna known from Britain from the last glacial period (Yalden 1999; Currant & Jacobi 2001). Fourth, *M. arvalis* is not currently found in Britain (Fig. 2). It is therefore contrary to think that *M. arvalis* should be a glacial relict on Orkney rather than *M. agrestis,* when the latter occurs further north in Eurasia (beyond the 70th parallel) and is distributed throughout Britain, including on many offshore islands, while *M. arvalis* only occurs on Orkney. There have been no land connections between mainland Britain and Orkney after conditions ameliorated following the LGM (Yalden 1982), and hence why *M. agrestis* (and other wide‐ranging small mammals in Britain, such as common shrews *Sorex araneus* and bank voles *Myodes glareolus*) failed to colonize Orkney. If *M. arvalis* did not survive on Orkney itself during the last glacial period, the absence of the species in Britain means there are no grounds to suggest sweepstake colonization from there. It is conceivable that there could have been sweepstake colonization of Orkney from Doggerland, the landmass connecting Britain, the Low Countries and Denmark until about 8000 bp (Weninger *et al*. 2008) – but this would require the survival of small mammals on floating mats of vegetation over a substantial marine gap between Doggerland and Orkney.

Thus, human introduction is by far the most likely explanation for the occurrence of *M. arvalis* on Orkney. From the IMa and ABC dates, this introduction at about 5000 bp fits well with the earliest radiocarbon dates for archaeological *M. arvalis* from Neolithic contexts (5100 years old: Table 2) and the beginnings of the Neolithic culture on Orkney (5600 bp: Ritchie 2001; Schulting *et al*. 2010). Voles could have been brought to Orkney by Mesolithic hunter‐gatherers, as early as *c*. 9000 bp, but no vole remains have been found in the one excavated Mesolithic site on Orkney (Lee & Woodward 2009), in contrast to their abundance at Neolithic and later sites (Yalden 1999; Thaw *et al*. 2004).

If, as appears most likely, the voles were introduced by Neolithic settlers about 5000 bp, various other implications flow from our molecular data, which are of considerable archaeological interest.

First, the introduction implies long‐distance maritime travel by Neolithic people between continental Europe and Orkney, extending on findings from elsewhere (e.g. Broodbank 2006). Our study highlights the Belgian coastline as the most reasonable source of the Orkney voles on the basis of available genetic data. This suggests Neolithic cultural linkages between Belgium and Orkney, of worthwhile focus for future archaeological investigation. *Microtus arvalis* were not introduced successfully into mainland Britain, which is consistent with relatively direct transport to Orkney from the continental source area.

Second, if the introduction occurred about 5000 bp, then, because the tMRCA for the Orkney voles is so long (15 400 years), substantial numbers of female voles must have been introduced to explain the *cytb* variation observed in modern and archaeological Orkney voles. High genetic diversity is already evident in the 16 aDNA sequences dating to 4200 bp or earlier, separated by up to 10 mutations (Fig. 3 and Table S3, Supporting information) and which produce an estimate for the tMRCA (14 780 years; 95% CI of 4681–36 379 years) similar to that of the full ancient and modern data set.

To explain a substantial number of voles arriving accidentally on Orkney implies transport of plentiful grass livestock bedding/fodder in which the vole stowaways could have survived. This in turn may suggest the direct movement of livestock as part of the proposed Neolithic linkage between Belgium and Orkney.

Alternatively, deliberate transport of voles onto Orkney could explain the large numbers introduced (Thaw *et al*. 2004). It is conceivable that voles were taken as food items, pets or for cultural/religious purposes – *M. arvalis* is docile in captivity (Berry 2000), so could theoretically have been ‘tamed’. This suggestion of deliberate transportation of small rodents has a precedent: it has been argued that Pacific rats (*Rattus exulans*), now present on islands throughout Oceania, were intentionally conveyed by Polynesians as a food source (Matisoo‐Smith & Robins 2004).

### Evolutionary processes affecting voles on Orkney and the continental source area

Compared with continental European *M. arvalis*, those on Orkney and another offshore island (Guernsey) are divergent in terms of tooth morphology, including increased tooth size. For Orkney, this divergence may have occurred over *c*. 5000 years, if introduced during the Neolithic. In addition to having larger teeth, the Orkney and Guernsey *M. arvalis* have a larger body size than continental voles (Gorman & Reynolds 2008). Quick‐evolving rodent gigantism has been described previously on islands of the northeast Atlantic (Corbet 1961; Angerbjorn 1986), but not within such a precisely defined time frame. A range of selective factors have been proposed to explain this gigantism, including an absence of small mammalian predators (Lomolino 1985), and a genetic basis for gigantism has been identified in island house mice (Chan *et al*. 2012). Elsewhere, we further explore the dynamics of morphological evolution for the Orkney *M. arvalis* using archaeological specimens (T. Cucchi, R. Barnett, N. Martínková, *et al*. submitted) extending substantially on previous studies (Berry & Rose 1975; Corbet 1986).

Despite their similarity in large tooth and body size, Guernsey and Orkney voles exhibit distinctive mtDNA haplotypes (Fig. 3). It is therefore most reasonable to consider that the Guernsey and Orkney voles attained their large body size independently. It is not clear whether Guernsey was colonized naturally before it became an island, as part of the continental European late glacial/postglacial species expansion (Haynes *et al*. 2003; Heckel *et al*. 2005; Tougard *et al*. 2008), or whether the voles were introduced by people after it became an island (Gorman & Reynolds 2008). However, given that Guernsey voles are likely to come from the same general (northern France/Belgium) source area as the Orkney voles and that they are also different in mtDNA from current northern France/Belgium populations, there would be much interest in further detailed comparison of Orkney, Guernsey and northern France/Belgium voles.

In addition to the operation of selection in the evolution of Orkney voles suggested by morphology, stochastic processes appear to have been important based on microsatellites. The population on the largest island, Mainland Orkney, has retained much of the microsatellite variation found in continental Europe, while all the other Orkney Islands (which are considerably smaller: Fig. 1) show very low levels of microsatellite variation, consistent with founder events and genetic drift. Similar stochastic processes can also explain microsatellite variation among Scottish Island populations of common shrew (White & Searle 2007a).

Our findings with regard to morphology and microsatellites in *M. arvalis* are unsurprising in comparison with previous studies on island small mammals, but the results from our mtDNA analyses are more unexpected. Although the *cytb* sequences from Orkney and the proposed source area for the Orkney colonization both belong to the Western‐North lineage of *M. arvalis*, the sequences are remarkably divergent given the time frame for colonization. Also, it might have been expected that (as for the microsatellites) variability would have been lower on Orkney than in continental Europe. In fact, the opposite is the case. Taking either the principal island (Mainland Orkney) or the whole archipelago, mtDNA diversity is higher in Orkney than in coastal France/Belgium (Table 3). Our dating analysis also shows that the mtDNA sequences in coastal France/Belgium have a much more recent derivation than the Orkney sequences.

So, here we are seeing another facet of evolution in association with the colonization of offshore islands, in this case occurring in the mainland population. The presence of derived sequences in coastal France/Belgium suggests a replacement event in *M. arvalis*, with one mtDNA type (the current type) replacing another (the Orkney type), similar to aDNA findings in other species (Barnes *et al*. 2002; Pergams *et al*. 2003; Hofreiter *et al*. 2007). The fact that there is an affiliation between coastal Belgium and Orkney on the basis of microsatellite genotypes argues against a complete population replacement (e.g. by extinction–recolonization) as an explanation for the mtDNA result. Instead, within‐population processes of selective sweeps or genetic drift are implicated, and more likely expressed in the mtDNA data, as a single locus with small effective population size than in the microsatellite data. We cannot be sure what environmental factors promoted the replacement. There could, for instance, have been a local, unrecorded disease outbreak. However, it is notable that the replacement occurred over a period when *M. arvalis* populations would have changed dramatically due to human land‐use change, and this appears the most likely driver of the replacement. Over several thousand years, sustained forest clearance in continental Europe (Rackham 1998; Cyprien *et al*. 2004) created new agricultural habitats and associated selection pressures that essentially expanded the opportunities for *M. arvalis* as a species that particularly exploits managed grassland (Niethammer & Krapp 1982). In such a habitat, *M. arvalis* populations can undergo massive population expansions and crashes (Delattre *et al*. 1992) that reduce long‐term effective population size, promoting genetic change through drift. On Orkney (which saw the rapid decline of low shrubs and tree species with the arrival of Neolithic farmers: Bunting 1996), *M. arvalis* utilizes a range of open habitats and does not show the same dramatic population fluctuations as seen in parts of continental Europe (Gorman & Reynolds 2008).

### Offshore islands as field laboratories

There has been a tendency to view offshore island populations of small mammals (and other organisms with low density and low dispersal) as genetic deviants from the ‘norm’. This is because studies of various species have shown results similar to ours for morphology (substantial change) and microsatellites (loss of variation) (Lomolino 1985; Frankham 1997; Boessenkool *et al*. 2007; Millien 2011). These have included detailed studies on small mammals such as wood mouse *Apodemus sylvaticus* (Angerbjorn 1986; Michaux *et al*. 1996), masked shrew *Sorex cinereus* (Stewart & Baker 1992) and common shrew (White & Searle 2007a,b, 2008).

However, as we have demonstrated with our *M. arvalis* mtDNA studies, island populations can also represent genetic ‘arks’, retaining the ancestral genetic variation, while evolutionary and other processes on the mainland may lead to a loss of that ancestral variation. Islands may have importance therefore in conservation of genetic variation. A further example involving human introduction of a small mammal onto an offshore island is provided by the Eurasian red squirrel *Sciurus vulgaris*. Thus, Irish red squirrels have genetic variants that apparently derive by introduction from Britain, but these are now absent in that source population (Finnegan *et al*. 2008; Searle 2008). For low density and low dispersal organisms such as small mammals, we suggest that genetic surveys of mainland areas should, where available, include populations from neighbouring offshore islands. It is very likely that those island populations will provide a new perspective on the temporal and spatial dynamics of the genetic variation in that region.

The ‘ark’ concept that we discuss here is of course more general. Populations colonizing new areas will take the genetic and nongenetic characteristics of the source population, and some of those characteristics may subsequently be lost in the source population but retained in the population in the new area. In this way, for instance, the United States is a ‘linguistic ark’ for various English words that would have been common in the British Isles at the time of settlement of North America by the British, but which have subsequently fallen into disuse in the homeland (e.g. ‘fall’ meaning ‘autumn’).

Returning to genetic characteristics of offshore islands, in addition to their potential as genetic ‘arks’, they also hold potential as field laboratories to study genetic change in the islands themselves. Compared with the classic evolutionary studies on oceanic islands, those based on offshore islands will tend to view events over shorter timescales and thus provide a different perspective on evolutionary processes. Offshore islands are particularly valuable for studying initial stages of diversification, with the opportunity (as in the current study together with T. Cucchi, R. Barnett, N. Martínková, *et al*. submitted) to follow island populations from their foundation to the present day using advanced genetic and morphometric tools as applied to modern and ancient populations of different ages. Extremely accurate dating of ancient populations may be possible (e.g. in archaeological settings). With this short time duration and close proximity to the mainland, there is also a greater chance to find the precise source area for the island colonization, which allows interesting comparison of evolutionary processes on the mainland and island. This brings us back to the value of offshore island populations in interpreting mainland processes. Offshore islands are an underutilized resource for evolutionary analysis, with great potential. In some ways, they represent study systems intermediate between those in a continental setting and those on oceanic islands; they have the simplicity of the oceanic island system yet are clearly relevant to continental situations.

K.M.D. and J.B.S. conceived, initiated and coordinated the project. T.C., M.F., G.H., N.M., M.P., Ma.P., J.‐P.Q. and J.B.S. organized and collected field specimens, R.B., T.C. and K.M.D. organized and obtained museum and archaeological samples. R.B., T.C. and N.M. conducted laboratory work supported by K.M.D., A.R.H., G.H. and J.B.S. and with major contributions from S.B. and T.H. R.B., T.C., N.M. and R.S. analysed the data supported by K.M.D., L.E., P.O'H., A.R.H., G.H., S.Y.W.H. and J.B.S. J.B.S. led the writing of the text to which all authors contributed.

## Data accessibility

The DNA sequences have been deposited in GenBank (GU190383‐GU190665). The TreeBASE entry for the phylogenetic tree of *cytb* sequences shown in Fig. 2, the microsatellite genotypes, the input file for the IMa analysis, the modern and ancient *cytb* sequences, and the morphological coordinates collected from 2D images of skulls are all available through DRYAD:

doi:10.5061/dryad.9rf5m.

## Supplementary Material

**Data S1** Material and Methods.
**Table S1** List of modern *M. arvalis* samples used for morphometrics, including country and site of origin.
**Table S2** List of all modern *M. arvalis* used for *cytb* analysis and collected for this study, including country and site of origin; arranged according to *cytb* haplotype.
**Table S3** List of all ancient specimens of *M. arvalis* that successfully provided a *cytb* sequence with details of location collected, calibrated age range (where obtained) and GenBank Accession Number for the sequence.
**Table S4** List of primers used for the amplification of cytochrome *b* from *M. arvalis*.
**Table S5** Prior distributions of the ABC model parameters (as illustrated in Fig. S3).
**Table S6** Pairwise *F*_ST_ values between population samples analysed with microsatellites (see Table 1 and Fig. S1).
**Fig. S1** Map showing distribution of population samples of modern *M. arvalis* used for microsatellite typing, labelled for mtDNA lineage. Population names as listed in Table 1: 1 – Heerenveen, 2 – Dinteloord, 3 – Stalhille, 4 – Veurne, 5 – Pihen lès Guînes, 6 – Fressenneville, 7 – Daubeuf, 8 – Thaon, 9 – Ste Marie du Mont, 10 – St Jean du Thomas, 11 – Baie d'Aiguillon, 12 – Aiffres, 13 – Avallon, 14 – Clérmont‐Ferrand, 15 – Alflen, 16 – Schiltach, 17 – Loch of Swartmill, 18 – Ness, 19 – Whitemill Bay, 20 – Settiscarth, 21 – Harray Stenness, 22 – St Ola, 23 – Grimness, 24 – Wind Wick.
**Fig. S2** Map showing Orkney localities where ancient specimens of *M. arvalis* successfully provided either radiocarbon dates and/or *cytb* sequences. Localities as listed in Tables 2 and S3: 1 ‐ Holm of Papa Westray, Westray, 2 ‐ Point of Cott, Westray, 3 ‐ Pierowall Quarry, Westray, 4 ‐ Quanterness, Mainland, 5 ‐ Earl's Bu, Mainland, 6 ‐ Howe, Mainland, 7 ‐ Skara Brae, Mainland, 8 ‐ Green Hill, South Walls, Hoy.
**Fig. S3** Diagram illustrating the ABC model parameters.
