# Supplementary material for: Divergent evolutionary processes associated with colonization of offshore islands
Source: Mol Ecol. 2013 Sep 3;22(20):5205–20. doi: 10.1111/mec.12462 (PMC4159590; doi:10.1111/mec.12462)
Supplement: Supplementary file 1 — Data S1 Material and Methods. Table S1 List of modern M. arvalis samples used for morphometrics, including country and site of origin. Table S2 List of all modern M. arvalis used for cytb analysis and collected for this study, including country and site of origin; arranged according to cytb haplotype. Table S3 List of all ancient specimens of M. arvalis that successfully provided a cytb sequence with details of location collected, calibrated age range (where obtained) and GenBank Accession Number for the sequence. Table S4 List of primers used for the amplification of cytochrome b from M. arvalis. Table S5 Prior distributions of the ABC model parameters (as illustrated in Fig. S3). Table S6 Pairwise FST values between population samples analysed with microsatellites (see Table 1 and Fig. S1). Fig. S1 Map showing distribution of population samples of modern M. arvalis used for microsatellite typing, labelled for mtDNA lineage. Population names as listed in Table 1: 1 – Heerenveen, 2 – Dinteloord, 3 – Stalhille, 4 – Veurne, 5 – Pihen lès Guînes, 6 – Fressenneville, 7 – Daubeuf, 8 – Thaon, 9 – Ste Marie du Mont, 10 – St Jean du Thomas, 11 – Baie d'Aiguillon, 12 – Aiffres, 13 – Avallon, 14 – Clérmont‐Ferrand, 15 – Alflen, 16 – Schiltach, 17 – Loch of Swartmill, 18 – Ness, 19 – Whitemill Bay, 20 – Settiscarth, 21 – Harray Stenness, 22 – St Ola, 23 – Grimness, 24 – Wind Wick. Fig. S2 Map showing Orkney localities where ancient specimens of M. arvalis successfully provided either radiocarbon dates and/or cytb sequences. Localities as listed in Tables 2 and S3: 1 ‐ Holm of Papa Westray, Westray, 2 ‐ Point of Cott, Westray, 3 ‐ Pierowall Quarry, Westray, 4 ‐ Quanterness, Mainland, 5 ‐ Earl's Bu, Mainland, 6 ‐ Howe, Mainland, 7 ‐ Skara Brae, Mainland, 8 ‐ Green Hill, South Walls, Hoy. Fig. S3 Diagram illustrating the ABC model parameters. [file mec-22-5205-s4.docx]

**Supporting Information**

**Martínková et al.**

**Data S1**

**Material and Methods**

*Approximate Bayesian Computation*

The ABC approach we took was as follows. We first carried out a large number of genetic simulations under a particular demographic and mutation model using the program SIMCOAL2 version 2.1.3 (Excoffier *et al.* 2000; Laval & Excoffier 2004). For each simulation, demographic and mutation parameters were randomly drawn from specified prior distributions. These parameters were used by the program SIMCOAL2 to generate genetic diversity for the same number of loci and individuals as in the observed data set. Going backward in time, SIMCOAL2 first reconstructs the genealogy of a sample of genes under the demographic history defined in the model, and then generates genetic data according to the sampled mutation parameters. The resulting simulated data were then described by the same set of summary statistics as calculated for the observed data. The next step was to compare the summary statistics of the simulated data with those of the observed data set by computing Euclidean distances. The 5,000 simulations with the smallest Euclidean distances (i.e. closest to the observed data) were retained for parameter estimation. In the estimation step, a multiple and locally weighted linear regression was used to approximate the posterior distributions of the parameters. From these posterior distributions, point estimates of the parameters such as the mode or median were obtained. Here, we used the program abcEst (Excoffier *et al*. 2005) to perform the last two steps. The program includes a transformation of the parameters before the regression as y = log[tan(x)^-1^] to guarantee that the posterior distributions remain within the range of the priors (Hamilton *et al*. 2005). Following Neuenschwander *et al.* (2008), we computed the coefficient of determination (R^2^) of the parameters by the statistics over all simulations as an index of the potential of a parameter to be correctly estimated. Values of R^2^ higher than 10% suggest that the summary statistics explain enough of the variability of a parameter to be correctly estimated in most cases (Neuenschwander 2006).

In order to simulate genetic diversity at microsatellite loci, we used a Generalized Stepwise Mutation (GSM) model (Di Rienzo *et al.* 1994; Kimmel & Chakraborty 1996) allowing mutations of more than one step to occur, according to an average mutation rate $\overline{\mu}$ and the geometric distribution parameter p. In this mutation model, microsatellite loci were considered unlinked, with locus-specific mutation rates following a Gamma distribution with a mean equal to the average mutation rate $\overline{\mu}$ but with varying shape depending on a parameter α. Based on the model described above, we performed 1 million simulations of the Orkney colonization, each of them generating microsatellite data of two samples corresponding to a continental European population and an Orkney population.

The prior distributions of all 14 parameters used to describe our model are defined in Table S5 (see also Fig. S3). They were specified by incorporating information available from the literature whenever possible and are justified below. Note that effective population sizes are expressed in number of haploid individuals and that times are measured in number of generations.

The oldest remains of *M. arvalis* in the fossil record of Orkney date back to approximately 5,000 years ago (Table 2). Therefore, the prior of the time of the Orkney colonization event (TOC) was set uniformly between 1,000 and 25,000 generations to provide a wide range of possibilities based on different colonization scenarios and possible numbers of generations per year (Table S5). The duration of the colonization bottleneck (dOC) was drawn randomly from a distribution between 5 and 100 generations. This range is probably not too narrow as the common vole is a prolific species (Hausser 1995), thus able to reach large population sizes in a few generations. The same prior range was used for the duration of the other bottleneck events (dB). Because there is no information about the effective size of the Orkney founding population (NOC), we chose a relatively wide prior, allowing values between 4 and 500. The same prior was also used for the effective population size during the bottlenecks in continental Europe and Orkney (NB). As a simplification, these two bottlenecks were determined using the same parameters regarding the duration of the bottleneck and the effective population size. The time of these two bottlenecks (TOB, TCB) were chosen independently using the same prior as for the colonization bottleneck, but with a more recent lower boundary of 110 instead of 1,000 for TOB as compared to TCB. Note that information about current population sizes of *M. arvalis* are highly variable in the literature and mainly based on census size. Thus, prior ranges for ancestral and current effective population sizes (NOA, NOR, NCA, NCR) were chosen based on our previous experience with the species (Heckel *et al*. 2005; Schweizer *et al*. 2007) as listed in Table S5.

The prior of the average mutation rate $\overline{\mu}$ was chosen between 5 x 10^-5^ and 7 x 10^-4^, including the often applied value of 5 x 10^-4^ for mammals (Ellegren 2004). Locus-specific mutation rates were then drawn from a gamma distribution Gamma (α, α/$\overline{\mu}$) where the shape parameter α was itself drawn from a uniform prior between 2 and 20. The difference in allele size upon each mutation was drawn from a geometric distribution with parameter p allowed to vary uniformly between 0 and 0.2. Here, p represents the fraction of cases where a mutation leads to a new allele size differing by more than one repeat from the original size.

To compare summary statistics, we used the same sample sizes for the simulated and observed data sets. Sample sizes of 19 and 20 individuals each were chosen for Orkney and continental European populations, respectively. Thus, for observed samples with a larger size, a new sample was created by randomly choosing 19 Orkney or 20 continental individuals. The random choice of individuals had very little effect on the variability of the summary statistics (results not shown).

We assessed the most likely source population of the Orkney voles by using a goodness of fit approach. Assuming that one of the sampled continental European populations is closest to the source of the Orkney voles (and the other populations are not), our model should best fit the observed genetic data for comparisons between Orkney and this population. For each of the 36 pairwise comparisons, we therefore used the modal point estimates of the parameters (instead of prior distributions) to generate 1,000 data sets under our model. The same set of summary statistics as used for the observed data was computed for each of these data sets. We then calculated the average distance ∆*_C_* between the observed and the simulated summary statistics of the three Orkney populations for each of the twelve continental populations as

;

where *P_O_* is the number of Orkney populations, *S* is the number of simulations, *ss* is the number of summary statistics, and *ss_obs_* is the observed summary statistic. Thus, the most likely source population is the one with the smallest ∆*_C_*, i.e. the continental population for which our model can best reproduce the observed genetic data for all three Orkney mainland populations.

Di Rienzo A, Peterson AC, Garza JC*, et al.* (1994) Mutational processes of simple-sequence repeat loci in human populations. *Proceedings of the National Academy of Sciences U S A* **91**, 3166-3170.

Ellegren H (2004) Microsatellites: simple sequences with complex evolution. *Nature Reviews Genetics* **5**, 435-445.

Excoffier L, Estoup A, Cornuet JM (2005) Bayesian analysis of an admixture model with mutations and arbitrarily linked markers. *Genetics* **169**, 1727-1738.

Excoffier L, Novembre J, Schneider S (2000) SIMCOAL: a general coalescent program for the simulation of molecular data in interconnected populations with arbitrary demography. *Journal of Heredity* **91**, 506-509.

Hamilton G, Stoneking M, Excoffier L (2005) Molecular analysis reveals tighter social regulation of immigration in patrilocal populations than in matrilocal populations. *Proceedings of the National Academy of Sciences USA* **102**, 7476-7480.

Hausser J (1995) *Säugetiere der Schweiz: Verbreitung, Biologie, Oekologie.* Birkhäuser Verlag, Basel.

Heckel G, Burri R, Fink S, Desmet JF, Excoffier L (2005) Genetic structure and colonization processes in European populations of the common vole, *Microtus arvalis*. *Evolution* **59**, 2231-2242.

Kimmel M, Chakraborty R (1996) Measures of variation at DNA repeat loci under a general stepwise mutation model. *Theoretical Population Biology* **50**, 345-367.

Laval G, Excoffier L (2004) SIMCOAL 2.0: a program to simulate genomic diversity over large recombining regions in a subdivided population with a complex history. *Bioinformatics* **20**, 2485-2487.

Neuenschwander S (2006) *Reconstruction of the post-glacial recolonization of the Swiss Alps by the bullhead (*Cottus gobio*) based on spatially explicit computer simulations.*, University of Bern.

Neuenschwander S, Largiader CR, Ray N*, et al.* (2008) Colonization history of the Swiss Rhine basin by the bullhead (*Cottus gobio*): inference under a Bayesian spatially explicit framework. *Molecular Ecology* **17**, 757-772.

Schweizer M, Excoffier L, Heckel G (2007) Fine-scale genetic structure and dispersal in the common vole (*Microtus arvalis*). *Molecular Ecology* **16**, 2463-2473.

**Table S1** List of modern *M. arvalis* samples used for morphometrics, including country and site of origin

| Collection location | Island | Region | Country | N | Collector/Source |
| --- | --- | --- | --- | --- | --- |
| Ezpezel |  | Aude | France | 30 | J. P. Quéré |
| Caen |  | Calvados | France | 29 | Ma. Pascal/E. Jones/T. Cucchi |
| Calais |  | Nord Pas-de-Calais | France | 23 | Ma. Pascal/ N. Martínková |
| Various |  | Cantal | France | 27 | MNHN Paris |
| Various |  | Côte d’or | France | 24 | Smithsonian Institution |
| Various |  | Haute-Savoie | France | 19 | MNHN Paris |
| Various |  | Ile de France | France | 17 | J. P. Quéré |
| Various |  | Loire-Atlantique | France | 12 | MNHN Paris |
| Ferel |  | Morbihan | France | 13 | J. P. Quéré |
|  | Noirmoutier | Vendée | France | 30 | J. P. Quéré |
| Mt Saint-Michel |  | Basse Normandie | France | 8 | Ma. Pascal/E. Jones |
| Saint-Michel en l'Hern |  | Vendée | France | 19 | J. P. Quéré |
| Le Magneraud |  | Vienne | France | 13 | J. P. Quéré |
|  | Yeu |  | France | 8 | Ma. Pascal/V. Bretille |
|  | Guernsey | Channel Islands | UK | 35 | BMNH London |
|  |  | Lower Saxony | Germany | 23 | Smithsonian Institution |
| Schiltach |  | Baden-Württemberg | Germany | 17 | G. Heckel/N. Martínková |
| Various |  |  | Hungary | 24 | BMNH London |
| Various |  |  | Spain | 15 | BMNH London |
| Various |  |  | Italy | 6 | BMNH London |
| Various |  |  | Ex-Yugoslavia | 30 | BMNH London |
| Various | Burray | Orkney | UK | 2 | N. Martínková/S. Martínek |
| Various | Mainland | Orkney | UK | 19 | N. Martínková/S. Martínek/G. Heckel |
|  |  |  |  | 27 | BMNH London/NMScotland |
| Various | Rousay | Orkney | UK | 1 | N. Martínková/S. Martínek |
|  |  |  |  | 9 | BMNH London/NMScotland |
| Various | Sanday | Orkney | UK | 3 | N. Martínková/S. Martínek |
|  |  |  |  | 17 | BMNH London/NMScotland |
| Various | Shapinsay | Orkney | UK | 3 | BMNH London/NMScotland |
| Various | S Ronaldsay | Orkney | UK | 15 | N. Martínková/S. Martínek |
|  |  |  |  | 2 | BMNH London/NMScotland |
| Various | Westray | Orkney | UK | 10 | N. Martínková/S. Martínek/G. Heckel |
|  |  |  |  | 23 | BMNH London/NMScotland |

**Table S2** List of all modern *M. arvalis* used for *cytb* analysis and collected for this study, including country and site of origin; arranged according to *cytb* haplotype

| Collection location | Island | Region | Country | Lat. | Long. | Collector/Source | Lineage | Haplotype | Acc. No. |
| --- | --- | --- | --- | --- | --- | --- | --- | --- | --- |
| Dinteloord |  |  | Netherlands | 51.64 | 4.37 | M. Fischer | Central | CEN1 | GU190526 |
| Dinteloord |  |  | Netherlands | 51.64 | 4.37 | M. Fischer | Central | CEN1 | GU190527 |
| Dinteloord |  |  | Netherlands | 51.64 | 4.37 | M. Fischer | Central | CEN1 | GU190528 |
| Dinteloord |  |  | Netherlands | 51.64 | 4.37 | M. Fischer | Central | CEN1 | GU190529 |
| Dinteloord |  |  | Netherlands | 51.64 | 4.37 | M. Fischer | Central | CEN1 | GU190530 |
| Nieuw-Vennep |  |  | Netherlands | 52.26 | 4.63 | M. Fischer | Central | CEN1 | GU190518 |
| Nieuw-Vennep |  |  | Netherlands | 52.26 | 4.63 | M. Fischer | Central | CEN1 | GU190521 |
| Pumerend |  |  | Netherlands | 52.51 | 4.94 | M. Fischer | Central | CEN1 | GU190513 |
| Pumerend |  |  | Netherlands | 52.51 | 4.94 | M. Fischer | Central | CEN1 | GU190514 |
| Pumerend |  |  | Netherlands | 52.51 | 4.94 | M. Fischer | Central | CEN1 | GU190515 |
| Pumerend |  |  | Netherlands | 52.51 | 4.94 | M. Fischer | Central | CEN1 | GU190516 |
| Middenmeer |  |  | Netherlands | 52.81 | 5.00 | M. Fischer | Central | CEN1 | GU190509 |
| Middenmeer |  |  | Netherlands | 52.81 | 5.00 | M. Fischer | Central | CEN1 | GU190510 |
| Middenmeer |  |  | Netherlands | 52.81 | 5.00 | M. Fischer | Central | CEN1 | GU190511 |
| Heerenveen |  |  | Netherlands | 52.96 | 5.93 | M. Fischer | Central | CEN2 | GU190499 |
| Heerenveen |  |  | Netherlands | 52.96 | 5.93 | M. Fischer | Central | CEN2 | GU190500 |
| Heerenveen |  |  | Netherlands | 52.96 | 5.93 | M. Fischer | Central | CEN2 | GU190501 |
| Heerenveen |  |  | Netherlands | 52.96 | 5.93 | M. Fischer | Central | CEN2 | GU190502 |
| Heerenveen |  |  | Netherlands | 52.96 | 5.93 | M. Fischer | Central | CEN2 | GU190503 |
| Wonseradeel |  |  | Netherlands | 53.09 | 5.44 | M. Fischer | Central | CEN2 | GU190504 |
| Wonseradeel |  |  | Netherlands | 53.09 | 5.44 | M. Fischer | Central | CEN2 | GU190505 |
| Wonseradeel |  |  | Netherlands | 53.09 | 5.44 | M. Fischer | Central | CEN2 | GU190506 |
| Nieuw-Vennep |  |  | Netherlands | 52.26 | 4.63 | M. Fischer | Central | CEN3 | GU190517 |
| Nieuw-Vennep |  |  | Netherlands | 52.26 | 4.63 | M. Fischer | Central | CEN3 | GU190519 |
| Nieuw-Vennep |  |  | Netherlands | 52.26 | 4.63 | M. Fischer | Central | CEN3 | GU190520 |
| Nieuw-Vennep |  |  | Netherlands | 52.26 | 4.63 | M. Fischer | Central | CEN3 | GU190522 |
| Pijnacker |  |  | Netherlands | 52.04 | 4.39 | M. Fischer | Central | CEN4 | GU190523 |
| Pijnacker |  |  | Netherlands | 52.04 | 4.39 | M. Fischer | Central | CEN4 | GU190524 |
| Pijnacker |  |  | Netherlands | 52.04 | 4.39 | M. Fischer | Central | CEN4 | GU190525 |
| NE Hamburg |  |  | Germany | 53.60 | 10.05 | J. Herman | Central | CEN5 | GU190662 |
| Nørre Farup |  |  | Denmark | 55.35 | 8.73 | J. Herman | Central | CEN6 | GU190660 |
| Nørre Farup |  |  | Denmark | 55.35 | 8.73 | J. Herman | Central | CEN7 | GU190661 |
| Middenmeer |  |  | Netherlands | 52.81 | 5.00 | M. Fischer | Central | CEN8 | GU190508 |
| Wonseradeel |  |  | Netherlands | 53.09 | 5.44 | M. Fischer | Central | CEN9 | GU190507 |
| Monthureux-le-Sec |  | Vosges | France | 48.17 | 6.03 | MNHN, Paris | Central | CEN10 | GU190390 |
| Pumerend |  |  | Netherlands | 52.51 | 4.94 | M. Fischer | Central | CEN11 | GU190512 |
| NE Hamburg |  |  | Germany | 53.60 | 10.05 | J. Herman | Central | CEN12 | GU190663 |
| Harray/Stenness | Mainland | Orkney | UK | 59.02 | -3.20 | N. Martínková/S. Martínek | Orkney | OrkneyA2 | GU190461 |
| Harray/Stenness | Mainland | Orkney | UK | 59.02 | -3.20 | N. Martínková/S. Martínek | Orkney | OrkneyA2 | GU190462 |
| Harray/Stenness | Mainland | Orkney | UK | 59.02 | -3.20 | C. Scott | Orkney | OrkneyA2 | GU190495 |
| Harray/Stenness | Mainland | Orkney | UK | 59.02 | -3.20 | C. Scott | Orkney | OrkneyA2 | GU190496 |
| Sandwick | Mainland | Orkney | UK | 59.10 | -3.29 | N. Martínková/S. Martínek | Orkney | OrkneyA2 | GU190478 |
| Sandwick | Mainland | Orkney | UK | 59.10 | -3.29 | N. Martínková/S. Martínek | Orkney | OrkneyA2 | GU190479 |
| Sandwick | Mainland | Orkney | UK | 59.10 | -3.29 | N. Martínková/S. Martínek | Orkney | OrkneyA2 | GU190480 |
| Sandwick | Mainland | Orkney | UK | 59.10 | -3.29 | N. Martínková/S. Martínek | Orkney | OrkneyA2 | GU190481 |
| Sandwick | Mainland | Orkney | UK | 59.10 | -3.29 | N. Martínková/S. Martínek | Orkney | OrkneyA2 | GU190482 |
| Little Sea | Sanday | Orkney | UK | 59.24 | -2.60 | N. Martínková/S. Martínek | Orkney | OrkneyC2 | GU190467 |
| Little Sea | Sanday | Orkney | UK | 59.24 | -2.60 | N. Martínková/S. Martínek | Orkney | OrkneyC2 | GU190468 |
| Little Sea | Sanday | Orkney | UK | 59.24 | -2.60 | N. Martínková/S. Martínek | Orkney | OrkneyC2 | GU190469 |
| Little Sea | Sanday | Orkney | UK | 59.24 | -2.60 | N. Martínková/S. Martínek | Orkney | OrkneyC2 | GU190470 |
| Little Sea | Sanday | Orkney | UK | 59.24 | -2.60 | N. Martínková/S. Martínek | Orkney | OrkneyC2 | GU190471 |
| Lady | Sanday | Orkney | UK | 59.26 | -2.54 | J. Herman | Orkney | OrkneyC2 | GU190403 |
| Whitemill Bay | Sanday | Orkney | UK | 59.30 | -2.55 | J. Herman | Orkney | OrkneyC2 | GU190397 |
| Whitemill Bay | Sanday | Orkney | UK | 59.30 | -2.55 | J. Herman | Orkney | OrkneyC2 | GU190398 |
| Whitemill Bay | Sanday | Orkney | UK | 59.30 | -2.55 | J. Herman | Orkney | OrkneyC2 | GU190399 |
| Whitemill Bay | Sanday | Orkney | UK | 59.30 | -2.55 | J. Herman | Orkney | OrkneyC2 | GU190400 |
| Whitemill Bay | Sanday | Orkney | UK | 59.30 | -2.55 | J. Herman | Orkney | OrkneyC2 | GU190401 |
| Whitemill Bay | Sanday | Orkney | UK | 59.30 | -2.55 | J. Herman | Orkney | OrkneyC2 | GU190402 |
| Whitemill Bay | Sanday | Orkney | UK | 59.30 | -2.55 | N. Martínková/S. Martínek | Orkney | OrkneyC2 | GU190466 |
| Southtown | Burray | Orkney | UK | 58.85 | -2.89 | N. Martínková/S. Martínek | Orkney | OrkneyD | GU190472 |
| Southtown | Burray | Orkney | UK | 58.85 | -2.89 | N. Martínková/S. Martínek | Orkney | OrkneyD | GU190473 |
| Ness | Westray | Orkney | UK | 59.24 | -2.87 | N. Martínková/S. Martínek | Orkney | OrkneyD | GU190427 |
| Ness | Westray | Orkney | UK | 59.24 | -2.87 | N. Martínková/S. Martínek | Orkney | OrkneyD | GU190428 |
| Ness | Westray | Orkney | UK | 59.24 | -2.87 | N. Martínková/S. Martínek | Orkney | OrkneyD | GU190429 |
| Ness | Westray | Orkney | UK | 59.24 | -2.87 | N. Martínková/S. Martínek | Orkney | OrkneyD | GU190430 |
| Ness | Westray | Orkney | UK | 59.24 | -2.87 | N. Martínková/S. Martínek/G. Heckel | Orkney | OrkneyD | GU190431 |
| Loch of Swartmill | Westray | Orkney | UK | 59.29 | -2.92 | N. Martínková/S. Martínek | Orkney | OrkneyD | GU190423 |
| Loch of Swartmill | Westray | Orkney | UK | 59.29 | -2.92 | N. Martínková/S. Martínek | Orkney | OrkneyD | GU190424 |
| Loch of Swartmill | Westray | Orkney | UK | 59.29 | -2.92 | N. Martínková/S. Martínek | Orkney | OrkneyD | GU190425 |
| Loch of Swartmill | Westray | Orkney | UK | 59.29 | -2.92 | N. Martínková/S. Martínek | Orkney | OrkneyD | GU190426 |
| Pierowall | Westray | Orkney | UK | 59.32 | -2.99 | N. Martínková/S. Martínek/G. Heckel | Orkney | OrkneyD | GU190475 |
| Pierowall | Westray | Orkney | UK | 59.32 | -2.99 | N. Martínková/S. Martínek/G. Heckel | Orkney | OrkneyD | GU190476 |
| Tankerness | Mainland | Orkney | UK | 58.96 | -2.86 | N. Martínková/S. Martínek | Orkney | ORK-O1 | GU190484 |
| Harray/Stenness | Mainland | Orkney | UK | 59.02 | -3.20 | N. Martínková/S. Martínek | Orkney | ORK-O1 | GU190460 |
| Harray/Stenness | Mainland | Orkney | UK | 59.02 | -3.20 | N. Martínková/S. Martínek | Orkney | ORK-O1 | GU190465 |
| Settiscarth | Mainland | Orkney | UK | 59.05 | -3.10 | N. Martínková/S. Martínek | Orkney | ORK-O1 | GU190449 |
| Settiscarth | Mainland | Orkney | UK | 59.05 | -3.10 | N. Martínková/S. Martínek | Orkney | ORK-O1 | GU190451 |
| Settiscarth | Mainland | Orkney | UK | 59.05 | -3.10 | N. Martínková/S. Martínek | Orkney | ORK-O1 | GU190454 |
| St. Ola | Mainland | Orkney | UK | 58.94 | -2.95 | N. Martínková/S. Martínek | Orkney | ORK-O2 | GU190455 |
| St. Ola | Mainland | Orkney | UK | 58.94 | -2.95 | N. Martínková/S. Martínek | Orkney | ORK-O2 | GU190456 |
| St. Ola | Mainland | Orkney | UK | 58.94 | -2.95 | N. Martínková/S. Martínek | Orkney | ORK-O2 | GU190457 |
| St. Ola | Mainland | Orkney | UK | 58.94 | -2.95 | N. Martínková/S. Martínek | Orkney | ORK-O2 | GU190458 |
| St. Ola | Mainland | Orkney | UK | 58.94 | -2.95 | N. Martínková/S. Martínek | Orkney | ORK-O2 | GU190459 |
| Tankerness | Mainland | Orkney | UK | 58.96 | -2.86 | N. Martínková/S. Martínek | Orkney | ORK-O2 | GU190483 |
| Settiscarth | Mainland | Orkney | UK | 59.05 | -3.10 | N. Martínková/S. Martínek | Orkney | ORK-O3 | GU190447 |
| Settiscarth | Mainland | Orkney | UK | 59.05 | -3.10 | N. Martínková/S. Martínek | Orkney | ORK-O3 | GU190448 |
| Settiscarth | Mainland | Orkney | UK | 59.05 | -3.10 | N. Martínková/S. Martínek | Orkney | ORK-O3 | GU190450 |
| Settiscarth | Mainland | Orkney | UK | 59.05 | -3.10 | N. Martínková/S. Martínek | Orkney | ORK-O3 | GU190452 |
| Settiscarth | Mainland | Orkney | UK | 59.05 | -3.10 | N. Martínková/S. Martínek | Orkney | ORK-O3 | GU190453 |
| Orphir | Mainland | Orkney | UK | 58.96 | -3.12 | N. Martínková/S. Martínek | Orkney | ORK-O4 | GU190494 |
| Stromness | Mainland | Orkney | UK | 58.98 | -3.27 | N. Wheale | Orkney | ORK-O4 | GU190490 |
| Harray/Stenness | Mainland | Orkney | UK | 59.02 | -3.20 | R. Marwick | Orkney | ORK-O4 | GU190491 |
| Harray/Stenness | Mainland | Orkney | UK | 59.02 | -3.20 | R. Marwick | Orkney | ORK-O4 | GU190492 |
| Harray/Stenness | Mainland | Orkney | UK | 59.02 | -3.20 | R. Marwick | Orkney | ORK-O4 | GU190493 |
| Stromness | Mainland | Orkney | UK | 58.98 | -3.27 | N. Wheale | Orkney | ORK-O5 | GU190488 |
| Stromness | Mainland | Orkney | UK | 58.98 | -3.27 | N. Wheale | Orkney | ORK-O5 | GU190489 |
| Stromness | Mainland | Orkney | UK | 58.98 | -3.27 | J. Herman | Orkney | ORK-O5 | GU190404 |
| Harray/Stenness | Mainland | Orkney | UK | 59.02 | -3.20 | N. Martínková/S. Martínek | Orkney | ORK-O6 | GU190463 |
| Harray/Stenness | Mainland | Orkney | UK | 59.02 | -3.20 | N. Martínková/S. Martínek | Orkney | ORK-O6 | GU190464 |
| Orphir | Mainland | Orkney | UK | 58.94 | -3.06 | N. Martínková/S. Martínek | Orkney | ORK-O7 | GU190477 |
| Wasbister | Rousay | Orkney | UK | 59.18 | -3.06 | N. Gould | Orkney | ORK-R1 | GU190485 |
| Wasbister | Rousay | Orkney | UK | 59.18 | -3.06 | N. Gould | Orkney | ORK-R1 | GU190486 |
| Wasbister | Rousay | Orkney | UK | 59.18 | -3.06 | N. Gould | Orkney | ORK-R1 | GU190487 |
| Wasbister | Rousay | Orkney | UK | 59.18 | -3.06 | N. Gould | Orkney | ORK-R1 | GU190497 |
| Wasbister | Rousay | Orkney | UK | 59.18 | -3.06 | N. Gould | Orkney | ORK-R1 | GU190498 |
| Rousay | Rousay | Orkney | UK | 59.18 | -3.06 | via J. Herman | Orkney | ORK-R1 | GU190651 |
| Rousay | Rousay | Orkney | UK | 59.18 | -3.06 | via J. Herman | Orkney | ORK-R1 | GU190652 |
| Newhouse | Rousay | Orkney | UK | 59.13 | -3.05 | N. Martínková/S. Martínek | Orkney | ORK-R2 | GU190474 |
| Rousay | Rousay | Orkney | UK | 59.18 | -3.06 | via J. Herman | Orkney | ORK-R2 | GU190650 |
| Wind Wick | South Ronaldsay | Orkney | UK | 58.76 | -2.94 | N. Martínková/S. Martínek | Orkney | ORK-SR1 | GU190440 |
| Wind Wick | South Ronaldsay | Orkney | UK | 58.76 | -2.94 | N. Martínková/S. Martínek | Orkney | ORK-SR1 | GU190441 |
| Wind Wick | South Ronaldsay | Orkney | UK | 58.76 | -2.94 | N. Martínková/S. Martínek | Orkney | ORK-SR1 | GU190442 |
| Wind Wick | South Ronaldsay | Orkney | UK | 58.76 | -2.94 | N. Martínková/S. Martínek | Orkney | ORK-SR1 | GU190443 |
| Wind Wick | South Ronaldsay | Orkney | UK | 58.76 | -2.94 | N. Martínková/S. Martínek | Orkney | ORK-SR1 | GU190444 |
| Wind Wick | South Ronaldsay | Orkney | UK | 58.76 | -2.94 | N. Martínková/S. Martínek | Orkney | ORK-SR1 | GU190445 |
| Wind Wick | South Ronaldsay | Orkney | UK | 58.76 | -2.94 | N. Martínková/S. Martínek | Orkney | ORK-SR1 | GU190446 |
| Grimness | South Ronaldsay | Orkney | UK | 58.82 | -2.91 | N. Martínková/S. Martínek/G. Heckel | Orkney | ORK-SR1 | GU190432 |
| Grimness | South Ronaldsay | Orkney | UK | 58.82 | -2.91 | N. Martínková/S. Martínek/G. Heckel | Orkney | ORK-SR1 | GU190433 |
| Grimness | South Ronaldsay | Orkney | UK | 58.82 | -2.91 | N. Martínková/S. Martínek/G. Heckel | Orkney | ORK-SR1 | GU190434 |
| Grimness | South Ronaldsay | Orkney | UK | 58.82 | -2.91 | N. Martínková/S. Martínek/G. Heckel | Orkney | ORK-SR1 | GU190435 |
| Grimness | South Ronaldsay | Orkney | UK | 58.82 | -2.91 | N. Martínková/S. Martínek | Orkney | ORK-SR1 | GU190437 |
| Grimness | South Ronaldsay | Orkney | UK | 58.82 | -2.91 | N. Martínková/S. Martínek | Orkney | ORK-SR1 | GU190438 |
| Grimness | South Ronaldsay | Orkney | UK | 58.82 | -2.91 | N. Martínková/S. Martínek | Orkney | ORK-SR2 | GU190436 |
| Wind Wick | South Ronaldsay | Orkney | UK | 58.76 | -2.94 | N. Martínková/S. Martínek | Orkney | ORK-SR3 | GU190439 |
| Loch of Swartmill | Westray | Orkney | UK | 59.29 | -2.92 | N. Martínková/S. Martínek | Orkney | ORK-W1 | GU190422 |
| Py, Vernet les Bains |  | Pyrenees | France | 42.50 | 2.35 | J. Michaux | Western-South | SpainB1 | GU190383 |
| Py, Vernet les Bains |  | Pyrenees | France | 42.50 | 2.35 | J. Michaux | Western-South | SpainB1 | GU190384 |
| Veurne |  |  | Belgium | 51.07 | 2.66 | M. Fischer | Western-North | WN1 | GU190541 |
| Veurne |  |  | Belgium | 51.07 | 2.66 | M. Fischer | Western-North | WN1 | GU190542 |
| Veurne |  |  | Belgium | 51.07 | 2.66 | M. Fischer | Western-North | WN1 | GU190543 |
| Veurne |  |  | Belgium | 51.07 | 2.66 | M. Fischer | Western-North | WN1 | GU190544 |
| Veurne |  |  | Belgium | 51.07 | 2.66 | M. Fischer | Western-North | WN1 | GU190545 |
| Stalhille |  |  | Belgium | 51.21 | 3.07 | M. Fischer | Western-North | WN1 | GU190540 |
| Cissé |  | Vienne | France | 46.64 | 0.23 | M. Pascal | Western-North | WN1 | GU190555 |
| Etrabonne |  | Doubs | France | 47.23 | 5.74 | MNHN, Paris | Western-North | WN1 | GU190393 |
| Cairon |  | Calvados | France | 49.24 | -0.46 | Ma. Pascal/E. Jones/T. Cucchi | Western-North | WN1 | GU190579 |
| Cairon |  | Calvados | France | 49.24 | -0.46 | Ma. Pascal/E. Jones/T. Cucchi | Western-North | WN1 | GU190581 |
| Cairon |  | Calvados | France | 49.24 | -0.46 | Ma. Pascal/E. Jones/T. Cucchi | Western-North | WN1 | GU190582 |
| Ste. Marie du Mont |  | Normandie | France | 49.38 | -1.23 | M. Pascal | Western-North | WN1 | GU190566 |
| Daubeuf |  | Seine-Maritime | France | 49.78 | 0.52 | Ma. Pascal/E. Jones | Western-North | WN1 | GU190590 |
| Daubeuf |  | Seine-Maritime | France | 49.78 | 0.52 | Ma. Pascal/E. Jones | Western-North | WN1 | GU190591 |
| Daubeuf |  | Seine-Maritime | France | 49.78 | 0.52 | Ma. Pascal/E. Jones | Western-North | WN1 | GU190592 |
| Daubeuf |  | Seine-Maritime | France | 49.78 | 0.52 | Ma. Pascal/E. Jones | Western-North | WN1 | GU190593 |
| Daubeuf |  | Seine-Maritime | France | 49.78 | 0.52 | Ma. Pascal/E. Jones | Western-North | WN1 | GU190594 |
| St. Valery sur Somme |  | Somme | France | 50.18 | 1.64 | Ma. Pascal/J. B. Searle | Western-North | WN1 | GU190599 |
| St. Valery sur Somme |  | Somme | France | 50.18 | 1.64 | Ma. Pascal/J. B. Searle | Western-North | WN1 | GU190600 |
| St. Valery sur Somme |  | Somme | France | 50.18 | 1.64 | Ma. Pascal/J. B. Searle | Western-North | WN1 | GU190601 |
| St. Valery sur Somme |  | Somme | France | 50.18 | 1.64 | Ma. Pascal/J. B. Searle | Western-North | WN1 | GU190602 |
| St. Valery sur Somme |  | Somme | France | 50.18 | 1.64 | Ma. Pascal/J. B. Searle | Western-North | WN1 | GU190603 |
| Escalles |  | Pas-de-Calais | France | 50.92 | 1.71 | Ma. Pascal/N. Martínková | Western-North | WN1 | GU190609 |
| Escalles |  | Pas-de-Calais | France | 50.92 | 1.71 | Ma. Pascal/N. Martínková | Western-North | WN1 | GU190610 |
| Coquelles |  | Pas-de-Calais | France | 50.93 | 1.80 | Ma. Pascal/N. Martínková | Western-North | WN1 | GU190611 |
| Coquelles |  | Pas-de-Calais | France | 50.93 | 1.80 | Ma. Pascal/N. Martínková | Western-North | WN1 | GU190612 |
| Coquelles |  | Pas-de-Calais | France | 50.93 | 1.80 | Ma. Pascal/N. Martínková | Western-North | WN1 | GU190613 |
| Luxemburg |  |  | Luxemburg | 49.61 | 6.13 | A. Frantz | Western-North | WN1 | GU190395 |
| Stalhille |  |  | Belgium | 51.21 | 3.07 | M. Fischer | Western-North | WN2 | GU190536 |
| Stalhille |  |  | Belgium | 51.21 | 3.07 | M. Fischer | Western-North | WN2 | GU190537 |
| Stalhille |  |  | Belgium | 51.21 | 3.07 | M. Fischer | Western-North | WN2 | GU190538 |
| Stalhille |  |  | Belgium | 51.21 | 3.07 | M. Fischer | Western-North | WN2 | GU190539 |
| Rots |  | Calvados | France | 49.20 | -0.47 | Ma. Pascal/E. Jones/T. Cucchi | Western-North | WN2 | GU190574 |
| Rots |  | Calvados | France | 49.20 | -0.47 | Ma. Pascal/E. Jones/T. Cucchi | Western-North | WN2 | GU190575 |
| Rots |  | Calvados | France | 49.20 | -0.47 | Ma. Pascal/E. Jones/T. Cucchi | Western-North | WN2 | GU190576 |
| Rots |  | Calvados | France | 49.20 | -0.47 | Ma. Pascal/E. Jones/T. Cucchi | Western-North | WN2 | GU190577 |
| Rots |  | Calvados | France | 49.20 | -0.47 | Ma. Pascal/E. Jones/T. Cucchi | Western-North | WN2 | GU190578 |
| Cairon |  | Calvados | France | 49.24 | -0.46 | Ma. Pascal/E. Jones/T. Cucchi | Western-North | WN2 | GU190580 |
| Cairon |  | Calvados | France | 49.24 | -0.46 | Ma. Pascal/E. Jones/T. Cucchi | Western-North | WN2 | GU190583 |
| Thaon |  | Calvados | France | 49.26 | -0.46 | Ma. Pascal/E. Jones/T. Cucchi | Western-North | WN2 | GU190584 |
| Thaon |  | Calvados | France | 49.26 | -0.46 | Ma. Pascal/E. Jones/T. Cucchi | Western-North | WN2 | GU190585 |
| Thaon |  | Calvados | France | 49.26 | -0.46 | Ma. Pascal/E. Jones/T. Cucchi | Western-North | WN2 | GU190586 |
| Thaon |  | Calvados | France | 49.26 | -0.46 | Ma. Pascal/E. Jones/T. Cucchi | Western-North | WN2 | GU190587 |
| Thaon |  | Calvados | France | 49.26 | -0.46 | Ma. Pascal/E. Jones/T. Cucchi | Western-North | WN2 | GU190588 |
| Clérmont-Ferrand |  | Puy-de-Dôme | France | 45.78 | 3.08 | G. Heckel | Western-North | WN3 | GU190627 |
| Otterswiller |  | Bas-Rhin | France | 48.73 | 7.38 | C. Tougard | Western-North | WN3 | GU190636 |
| Schiltach |  | Black Forrest | Germany | 48.30 | 8.34 | G. Heckel/N. Martínková | Western-North | WN3 | GU190618 |
| Schiltach |  | Black Forrest | Germany | 48.30 | 8.34 | G. Heckel/N. Martínková | Western-North | WN3 | GU190619 |
| Schiltach |  | Black Forrest | Germany | 48.30 | 8.34 | G. Heckel/N. Martínková | Western-North | WN3 | GU190620 |
| Schiltach |  | Black Forrest | Germany | 48.30 | 8.34 | G. Heckel/N. Martínková | Western-North | WN3 | GU190621 |
| Schiltach |  | Black Forrest | Germany | 48.30 | 8.34 | G. Heckel/N. Martínková | Western-North | WN3 | GU190622 |
| Wolfach |  | Black Forrest | Germany | 48.30 | 8.22 | G. Heckel/N. Martínková | Western-North | WN3 | GU190623 |
| Wolfach |  | Black Forrest | Germany | 48.30 | 8.22 | G. Heckel/N. Martínková | Western-North | WN3 | GU190624 |
| Wolfach |  | Black Forrest | Germany | 48.30 | 8.22 | G. Heckel/N. Martínková | Western-North | WN3 | GU190625 |
| Wolfach |  | Black Forrest | Germany | 48.30 | 8.22 | G. Heckel/N. Martínková | Western-North | WN3 | GU190626 |
| Guernsey | Guernsey |  | UK | 49.46 | -2.63 | Guernsey Museum | Western-North | WN4 | GU190653 |
| Guernsey | Guernsey |  | UK | 49.46 | -2.63 | Guernsey Museum | Western-North | WN4 | GU190655 |
| Guernsey | Guernsey |  | UK | 49.46 | -2.63 | Guernsey Museum | Western-North | WN4 | GU190656 |
| Guernsey | Guernsey |  | UK | 49.46 | -2.63 | Guernsey Museum | Western-North | WN4 | GU190657 |
| Guernsey | Guernsey |  | UK | 49.46 | -2.63 | Guernsey Museum | Western-North | WN4 | GU190658 |
| Guernsey | Guernsey |  | UK | 49.46 | -2.63 | Guernsey Museum | Western-North | WN4 | GU190659 |
| Oostburg |  |  | Netherlands | 51.33 | 3.49 | M. Fischer | Western-North | WN5 | GU190531 |
| Oostburg |  |  | Netherlands | 51.33 | 3.49 | M. Fischer | Western-North | WN5 | GU190532 |
| Oostburg |  |  | Netherlands | 51.33 | 3.49 | M. Fischer | Western-North | WN5 | GU190533 |
| Oostburg |  |  | Netherlands | 51.33 | 3.49 | M. Fischer | Western-North | WN5 | GU190534 |
| Oostburg |  |  | Netherlands | 51.33 | 3.49 | M. Fischer | Western-North | WN5 | GU190535 |
| St. Jean le Thomas |  | Manche | France | 48.73 | -1.51 | Ma. Pascal/V. Bretille | Western-North | WN6 | GU190571 |
| St. Jean le Thomas |  | Manche | France | 48.73 | -1.51 | Ma. Pascal/V. Bretille | Western-North | WN6 | GU190572 |
| St. Jean le Thomas |  | Manche | France | 48.73 | -1.51 | Ma. Pascal/V. Bretille | Western-North | WN6 | GU190573 |
| Alflen |  |  | Germany | 50.18 | 7.04 | B. Walther/G. Heckel | Western-North | WN7 | GU190614 |
| Alflen |  |  | Germany | 50.18 | 7.04 | B. Walther/G. Heckel | Western-North | WN7 | GU190615 |
| Alflen |  |  | Germany | 50.18 | 7.04 | B. Walther/G. Heckel | Western-North | WN7 | GU190617 |
| Fressenneville |  | Somme | France | 50.07 | 1.58 | Ma. Pascal/J. B. Searle | Western-North | WN8 | GU190595 |
| Fressenneville |  | Somme | France | 50.07 | 1.58 | Ma. Pascal/J. B. Searle | Western-North | WN8 | GU190596 |
| Fressenneville |  | Somme | France | 50.07 | 1.58 | Ma. Pascal/J. B. Searle | Western-North | WN8 | GU190598 |
| Pihen lès Guînes |  | Pas-de-Calais | France | 50.87 | 1.79 | Ma. Pascal/N. Martínková | Western-North | WN9 | GU190604 |
| Pihen lès Guînes |  | Pas-de-Calais | France | 50.87 | 1.79 | Ma. Pascal/N. Martínková | Western-North | WN9 | GU190605 |
| Pihen lès Guînes |  | Pas-de-Calais | France | 50.87 | 1.79 | Ma. Pascal/N. Martínková | Western-North | WN9 | GU190608 |
| Cissé |  | Vienne | France | 46.64 | 0.23 | M. Pascal | Western-North | WN10 | GU190553 |
| Cissé |  | Vienne | France | 46.64 | 0.23 | M. Pascal | Western-North | WN10 | GU190556 |
| Crépaillat |  | Le Quartier | France | 46.14 | 2.74 | M. Pascal | Western-North | WN11 | GU190405 |
| Crépaillat |  | Le Quartier | France | 46.14 | 2.74 | M. Pascal | Western-North | WN11 | GU190409 |
| Crépaillat |  | Le Quartier | France | 46.14 | 2.74 | M. Pascal | Western-North | WN12 | GU190406 |
| Crépaillat |  | Le Quartier | France | 46.14 | 2.74 | M. Pascal | Western-North | WN12 | GU190407 |
| Outre |  | Marcillat | France | 46.08 | 3.03 | M. Pascal | Western-North | WN13 | GU190413 |
| Outre |  | Marcillat | France | 46.08 | 3.03 | M. Pascal | Western-North | WN13 | GU190414 |
| Outre |  | Marcillat | France | 46.08 | 3.03 | M. Pascal | Western-North | WN14 | GU190415 |
| Outre |  | Marcillat | France | 46.08 | 3.03 | M. Pascal | Western-North | WN14 | GU190416 |
| Pihen lès Guînes |  | Pas-de-Calais | France | 50.87 | 1.79 | Ma. Pascal/N. Martínková | Western-North | WN15 | GU190606 |
| Pihen lès Guînes |  | Pas-de-Calais | France | 50.87 | 1.79 | Ma. Pascal/N. Martínková | Western-North | WN15 | GU190607 |
| Crépaillat |  | Le Quartier | France | 46.14 | 2.74 | M. Pascal | Western-North | WN16 | GU190408 |
| Outre |  | Marcillat | France | 46.08 | 3.03 | M. Pascal | Western-North | WN17 | GU190411 |
| Outre |  | Marcillat | France | 46.08 | 3.03 | M. Pascal | Western-North | WN18 | GU190412 |
| Téteghem |  | Nord | France | 51.02 | 2.44 | M. Fischer | Western-North | WN19 | GU190546 |
| Cissé |  | Vienne | France | 46.64 | 0.23 | M. Pascal | Western-North | WN20 | GU190552 |
| Cissé |  | Vienne | France | 46.64 | 0.23 | M. Pascal | Western-North | WN21 | GU190554 |
| Ste. Marie du Mont |  | Normandie | France | 49.38 | -1.23 | M. Pascal | Western-North | WN22 | GU190569 |
| Ste. Marie du Mont |  | Normandie | France | 49.38 | -1.23 | M. Pascal | Western-North | WN23 | GU190570 |
| Valmont |  | Seine-Maritime | France | 49.74 | 0.51 | Ma. Pascal/E. Jones | Western-North | WN24 | GU190589 |
| Fressenneville |  | Somme | France | 50.07 | 1.58 | Ma. Pascal/J. B. Searle | Western-North | WN25 | GU190597 |
| Alflen |  |  | Germany | 50.18 | 7.04 | B. Walther/G. Heckel | Western-North | WN26 | GU190616 |
| Clérmont-Ferrand |  | Puy-de-Dôme | France | 45.78 | 3.08 | G. Heckel | Western-North | WN27 | GU190628 |
| Wiwersheim |  | Bas-Rhin | France | 48.64 | 7.61 | C. Tougard | Western-North | WN28 | GU190635 |
| Guernsey | Guernsey |  | UK | 49.46 | -2.63 | Guernsey Museum | Western-North | WN29 | GU190654 |
| Etrabonne |  | Doubs | France | 47.23 | 5.74 | MNHN, Paris | Western-North | WN30 | GU190392 |
| Monthureux-le-Sec |  | Vosges | France | 48.17 | 6.03 | MNHN, Paris | Western-North | WN31 | GU190391 |
| Luxemburg |  |  | Luxemburg | 49.61 | 6.13 | A. Frantz | Western-North | WN32 | GU190396 |
| La Côte |  | Bussières | France | 46.07 | 2.66 | M. Pascal | Western-North | WN33 | GU190410 |
| Chenac-St Seurin d'Utz |  | Charente-Maritime | France | 45.52 | -0.83 | MNHN, Paris | Western-South | WS1 | GU190394 |
| Aiffres |  | Deux-Sèvres | France | 46.29 | -0.41 | M. Pascal | Western-South | WS1 | GU190547 |
| Aiffres |  | Deux-Sèvres | France | 46.29 | -0.41 | M. Pascal | Western-South | WS1 | GU190551 |
| Baie de l'Aiguillon |  |  | France | 46.30 | 1.17 | M. Pascal | Western-South | WS1 | GU190418 |
| Baie de l'Aiguillon |  |  | France | 46.30 | 1.17 | M. Pascal | Western-South | WS1 | GU190419 |
| Baie de l'Aiguillon |  |  | France | 46.30 | 1.17 | M. Pascal | Western-South | WS1 | GU190420 |
| Les Forts |  | Eure-et-Loir | France | 48.33 | 1.18 | M. Pascal | Western-South | WS1 | GU190558 |
| Les Forts |  | Eure-et-Loir | France | 48.33 | 1.18 | M. Pascal | Western-South | WS1 | GU190559 |
| Burgos |  | Burgos | Spain | 42.34 | -3.70 | J. Pauperio | Western-South | WS2 | GU190642 |
| Burgos |  | Burgos | Spain | 42.34 | -3.70 | J. Pauperio | Western-South | WS2 | GU190643 |
| Burgos |  | Burgos | Spain | 42.34 | -3.70 | J. Pauperio | Western-South | WS2 | GU190644 |
| Burgos |  | Burgos | Spain | 42.34 | -3.70 | J. Pauperio | Western-South | WS2 | GU190645 |
| Burgos |  | Burgos | Spain | 42.34 | -3.70 | J. Pauperio | Western-South | WS2 | GU190649 |
| Vega de Infanzones |  | León | Spain | 42.48 | -5.53 | J. Pauperio | Western-South | WS2 | GU190640 |
| Vega de Infanzones |  | León | Spain | 42.48 | -5.53 | J. Pauperio | Western-South | WS2 | GU190664 |
| Burgos |  | Burgos | Spain | 42.34 | -3.70 | J. Pauperio | Western-South | WS3 | GU190647 |
| Burgos |  | Burgos | Spain | 42.34 | -3.70 | J. Pauperio | Western-South | WS3 | GU190648 |
| Vega de Infanzones |  | León | Spain | 42.48 | -5.53 | J. Pauperio | Western-South | WS3 | GU190637 |
| Vega de Infanzones |  | León | Spain | 42.48 | -5.53 | J. Pauperio | Western-South | WS3 | GU190665 |
| Île d'Yeu | Yeu |  | France | 46.72 | -2.35 | Ma. Pascal/V. Bretille | Western-South | WS4 | GU190629 |
| Île d'Yeu | Yeu |  | France | 46.72 | -2.35 | Ma. Pascal/V. Bretille | Western-South | WS4 | GU190631 |
| Île d'Yeu | Yeu |  | France | 46.72 | -2.35 | Ma. Pascal/V. Bretille | Western-South | WS4 | GU190632 |
| Île d'Yeu | Yeu |  | France | 46.72 | -2.35 | Ma. Pascal/V. Bretille | Western-South | WS4 | GU190633 |
| Plà de Beret |  | Vall d'Aran | Spain | 42.72 | 0.84 | M. L. Fuster | Western-South | WS5 | GU190386 |
| Plà de Beret |  | Vall d'Aran | Spain | 42.72 | 0.84 | M. L. Fuster | Western-South | WS5 | GU190387 |
| Plà de Beret |  | Vall d'Aran | Spain | 42.72 | 0.84 | M. L. Fuster | Western-South | WS5 | GU190388 |
| Les Forts |  | Eure-et-Loir | France | 48.33 | 1.18 | M. Pascal | Western-South | WS6 | GU190557 |
| Les Forts |  | Eure-et-Loir | France | 48.33 | 1.18 | M. Pascal | Western-South | WS6 | GU190561 |
| Île de Oléron | Oléron |  | France | 46.00 | -1.35 | M. Pascal | Western-South | WS7 | GU190564 |
| Île de Oléron | Oléron |  | France | 46.00 | -1.35 | M. Pascal | Western-South | WS7 | GU190565 |
| Île de Oléron | Oléron |  | France | 46.00 | -1.35 | M. Pascal | Western-South | WS8 | GU190562 |
| Île de Oléron | Oléron |  | France | 46.00 | -1.35 | M. Pascal | Western-South | WS8 | GU190563 |
| Aiffres |  | Deux-Sèvres | France | 46.29 | -0.41 | M. Pascal | Western-South | WS9 | GU190549 |
| Les Forts |  | Eure-et-Loir | France | 48.33 | 1.18 | M. Pascal | Western-South | WS9 | GU190560 |
| Ste. Marie du Mont |  | Normandie | France | 49.38 | -1.23 | M. Pascal | Western-South | WS10 | GU190567 |
| Ste. Marie du Mont |  | Normandie | France | 49.38 | -1.23 | M. Pascal | Western-South | WS10 | GU190568 |
| Baie de l'Aiguillon |  |  | France | 46.30 | 1.17 | M. Pascal | Western-South | WS11 | GU190421 |
| Aiffres |  | Deux-Sèvres | France | 46.29 | -0.41 | M. Pascal | Western-South | WS12 | GU190548 |
| Aiffres |  | Deux-Sèvres | France | 46.29 | -0.41 | M. Pascal | Western-South | WS13 | GU190550 |
| Île d'Yeu | Yeu |  | France | 46.72 | -2.35 | Ma. Pascal/V. Bretille | Western-South | WS14 | GU190630 |
| Armendarits |  | Pyrénées-Atlantiques | France | 43.30 | -1.17 | C. Tougard | Western-South | WS15 | GU190634 |
| Vega de Infanzones |  | León | Spain | 42.48 | -5.53 | J. Pauperio | Western-South | WS16 | GU190638 |
| Vega de Infanzones |  | León | Spain | 42.48 | -5.53 | J. Pauperio | Western-South | WS17 | GU190639 |
| Vega de Infanzones |  | León | Spain | 42.48 | -5.53 | J. Pauperio | Western-South | WS18 | GU190641 |
| Burgos |  | Burgos | Spain | 42.34 | -3.70 | J. Pauperio | Western-South | WS19 | GU190646 |
| Plà de Beret |  | Vall d'Aran | Spain | 42.72 | 0.84 | M. L. Fuster | Western-South | WS20 | GU190385 |
| Plà de Beret |  | Vall d'Aran | Spain | 42.72 | 0.84 | M. L. Fuster | Western-South | WS21 | GU190389 |
| Baie de l'Aiguillon |  |  | France | 46.30 | 1.17 | M. Pascal | Western-South | WS22 | GU190417 |

**Table S3** List of all ancient specimens of *M. arvalis* that successfully provided a *cytb* sequence with details of location collected, calibrated age range (where obtained) and GenBank Accession Number for the sequence

| Specimen number | Locality | Island/ Country | Latitude | Longitude | 95.4 % (2s)  cal age ranges | Source* | Accession Number |
| --- | --- | --- | --- | --- | --- | --- | --- |
| R2 | Skara Brae | Mainland | 59.04 | -3.33 |  | NMS | GU197787 |
| R3 | Skara Brae | Mainland | 59.04 | -3.33 | cal BP 4294-4515 | NMS | GU197788 |
| R6 | Skara Brae | Mainland | 59.04 | -3.33 |  | NMS | GU197789 |
| R11 | Skara Brae | Mainland | 59.04 | -3.33 | cal BP 4529-4815 | NMS | GU197790 |
| R16 | Holm of Papa Westray | Westray | 59.35 | -2.86 |  | OM | GU197791 |
| R20 | Holm of Papa Westray | Westray | 59.35 | -2.86 | cal BP 4435-4784 | OM | GU197792 |
| R23 | Holm of Papa Westray | Westray | 59.35 | -2.86 | cal BP 4448-4808 | OM | GU197793 |
| R25 | Holm of Papa Westray | Westray | 59.35 | -2.86 |  | OM | GU197794 |
| R29 | Earl’s Bu | Mainland | 58.93 | -3.18 | cal BP 795-932 | MICU | GU197795 |
| R30 | Earl’s Bu | Mainland | 58.93 | -3.18 |  | MICU | GU197796 |
| R31 | Earl’s Bu | Mainland | 58.93 | -3.18 |  | MICU | GU197797 |
| R37 | Point of Cott | Westray | 59.28 | -2.89 | cal BP 4967-5288 | OM | GU197790 |
| R39 | Point of Cott | Westray | 59.28 | -2.89 |  | OM | GU197799 |
| R43 | Point of Cott | Westray | 59.28 | -2.89 |  | OM | GU197800 |
| R44 | Point of Cott | Westray | 59.28 | -2.89 | cal BP 5050-5437 | OM | GU197801 |
| R45 | Point of Cott | Westray | 59.28 | -2.89 | cal BP 4884-5287 | OM | GU197802 |
| R58 | Howe | Mainland | 58.98 | -3.33 | cal BP 1721-1869 | OM | GU197803 |
| R59 | Howe | Mainland | 58.98 | -3.33 | cal BP 1714-1865 | OM | GU197804 |
| R60 | Howe | Mainland | 58.98 | -3.33 |  | OM | GU197805 |
| R62 | Howe | Mainland | 58.98 | -3.33 | cal BP 1308-1396 | OM | GU197806 |
| R99 | Pierowall Quarry | Westray | 59.32 | -2.99 | cal BP 4092-4406 | OM | GU197807 |
| R124 | Pierowall Quarry | Westray | 59.32 | -2.99 | cal BP 4094-4406 | OM | GU197808 |
| R125 | Pierowall Quarry | Westray | 59.32 | -2.99 |  | OM | GU197809 |
| R126 | Pierowall Quarry | Westray | 59.32 | -2.99 | cal BP 4298-4781 | OM | GU197810 |
| R130 | Ninove | Belgium | 50.50 | 4.10 |  | FHI | GU197817 |
| R131 | Ninove | Belgium | 50.50 | 4.10 |  | FHI | GU197818 |
| R133 | Ninove | Belgium | 50.50 | 4.10 |  | FHI | GU197819 |
| R134 | Ninove | Belgium | 50.50 | 4.10 |  | FHI | GU197820 |
| R136 | Ninove | Belgium | 50.50 | 4.10 |  | FHI | GU197821 |
| R137 | Ninove | Belgium | 50.50 | 4.10 |  | FHI | GU197822 |
| R138 | Ninove | Belgium | 50.50 | 4.10 |  | FHI | GU197823 |
| R177 | Quanterness | Mainland | 58.59 | -3.0 | cal BP 4869-5257 | BMNH | GU197811 |
| R179 | Quanterness | Mainland | 58.59 | -3.0 | cal BP 4861-5213 | BMNH | GU197812 |
| R189 | Skara Brae | Mainland | 59.04 | -3.33 | cal BP 4185-4418 | NMS | GU197813 |
| R191 | Skara Brae | Mainland | 59.04 | -3.33 | cal BP 4622-4844 | NMS | GU197814 |
| R194 | Skara Brae | Mainland | 59.04 | -3.33 | cal BP 4256-4514 | NMS | GU197815 |
| R195 | Skara Brae | Mainland | 59.04 | -3.33 |  | NMS | GU197816 |

Individuals are from Mainland Orkney, Westray or Belgium and calibrated age ranges obtained as in Table 2. NMS = Jeremy Herman, National Museums of Scotland. BMNH = Richard Sabin, British Museum - Natural History, London. OM = Anne Brundle, The Orkney Museum. MICU = James Barrett, MacDonald Institute, University of Cambridge. FHI = Anton Ervynck, Flemish Heritage Institute.

**Table S4** List of primers used for the amplification of cytochrome *b* from *M. arvalis*

| Forward primer | | Reverse primer | | Ancient or modern DNA? | Annealing temp. °C | Fragment length in bp |
| --- | --- | --- | --- | --- | --- | --- |
| Name | Sequence 5’-3’ | Name | Sequence 5’-3’ |  |  |  |
| MaoF1 | AAACACCTAATGACAGTCATCC | MaoR1 | GATGAGAATGCTGTTGCTGTG | Ancient | 52 | 203 |
| MaoF2 | TTGTCTGATTGTCCARATTCTCA | MaoR2 | GTTATAGGAGCCGTAGTAGAC | Ancient | 48 | 208 |
| MaoF3 | ATACATGCCAACGGAGCTTCC | MaoR3 | CCTCAAATCCACTCTACTAGTG | Ancient | 48 | 254 |
| MaoF4 | GAGCCACAGTNATTACAAATCTC | MaoR4 | GTTGTTAGATCCTGTTTCGTGA | Ancient | 52 | 197 |
| MaoF5 | TTATCATTACCGCCCTCGTATT | MaoR5C | TTGTCGGGGTCTCCGAGAATATCTG | Ancient | 48 | 213 |
| MaoF5 | TTATCATTACCGCCCTCGTATT | MaoR6 | GGGATAGATCGTAGAATGGC | Ancient | 48 | 295 |
| MaoF6 | ATTATACAGTCAAAGATTTCCTAG | MaoR6 | GGGATAGATCGTAGAATGGC | Ancient | 52 | 187 |
| MaoF7 | TACACCCCTGCAAATCCACTC | MaoR7 | TGGGCGGAAAGTCAGTGCTC | Ancient | 52 | 192 |
| MaoF8 | CTAGCACTAATCCTATCAATCG | MaoR8 | CAATTATGCCTGCAATTGGTATG | Ancient | 52 | 244 |
| MaoF9 | AGCCAGTTGAATACCCATTCAT | H15915-SP | TTCATTACTGGTTTACAAGAC* | Ancient | 48 | 170 |
| L14727-SP | GACAGGAAAAATCATCGTTG* | H15915-SP | TTCATTACTGGTTTACAAGAC* | Modern | 52 | 1188 |
| L15162M2 | GCTACGTACTTCCATGAGGACAAATATC |  |  | Modern |  | Sequencing |
| H15348A-SP | GTTGGAYCCTGTTTCGTG |  |  | Modern |  | Sequencing |

*Jaarola, Searle (2002) Phylogeography of field voles (*Microtus agrestis*) in Eurasia inferred from mitochondrial DNA sequences. *Molecular Ecology,* **11**, 2613-2621.

**Table S5** Prior distributions of the ABC model parameters (as illustrated in Fig. S3)

| Parameter | Abbreviation | min | max |
| --- | --- | --- | --- |
|  |  |  |  |
| Effective population sizes |  |  |  |
| Ancestral size of continental population | NCA | 1,000 | 200,000 |
| Current size of continental population | NCR | 1,000 | 100,000 |
| Size during the colonization bottleneck | NOC | 4 | 500 |
| Size during the bottlenecks on the continent and on Orkney | NB | 4 | 500 |
| Ancestral size of Orkney population | NOA | 1,000 | 50,000 |
| Current size of Orkney population | NOR | 1,000 | 50,000 |
|  |  |  |  |
| Timing |  |  |  |
| Duration of the colonization bottleneck | dOC | 5 | 100 |
| Duration of the bottlenecks on the continent and on Orkney | dB | 5 | 100 |
| Time of the colonization of Orkney | TOC | 1,000 | 25,000 |
| Time of the Orkney bottleneck | TOB | 110 | 24,900 |
| Time of the continental bottleneck | TCB | 1,000 | 25,000 |
|  |  |  |  |
| Marker parameters |  |  |  |
| Mean mutation rate per generation | $\overline{\mu}$ | 5 x 10^-5^ | 7 x 10^-4^ |
| Parameter of the geometric distribution of mutation steps | p | 0 | 0.2 |
| Shape parameter for the gamma distribution of individual mutation rates | α | 2 | 20 |
|  |  |  |  |

All distributions are uniform between a minimum (min) and a maximum (max) value. Effective population sizes are in numbers of haploid individuals; times are reported in number of generations.

**Table S6** Pairwise F_ST_ values between population samples analysed with microsatellites (see Table 1 and Fig. S1)

|  | 1 | 2 | 3 | 4 | 5 | 6 | 7 | 8 | 9 | 10 | 11 | 12 |
| --- | --- | --- | --- | --- | --- | --- | --- | --- | --- | --- | --- | --- |
| 1. Heerenveen | 0 |  |  |  |  |  |  |  |  |  |  |  |
| 2. Dinteloord | 0.1631 | 0 |  |  |  |  |  |  |  |  |  |  |
| 3. Stalhille | 0.19569 | 0.22547 | 0 |  |  |  |  |  |  |  |  |  |
| 4. Veurne | 0.236 | 0.2986 | 0.15622 | 0 |  |  |  |  |  |  |  |  |
| 5. Pihen lès Guînes | 0.12983 | 0.19066 | 0.10297 | 0.15174 | 0 |  |  |  |  |  |  |  |
| 6. Fressenneville | 0.16974 | 0.21422 | 0.09083 | 0.1586 | 0.06645 | 0 |  |  |  |  |  |  |
| 7. Daubeuf | 0.15358 | 0.20526 | 0.08982 | 0.15826 | 0.04182 | 0.05777 | 0 |  |  |  |  |  |
| 8. Thaon | 0.17816 | 0.21383 | 0.11645 | 0.18865 | 0.06841 | 0.06816 | 0.07702 | 0 |  |  |  |  |
| 9. Ste Marie du Mont | 0.23918 | 0.29799 | 0.1969 | 0.24832 | 0.14118 | 0.16093 | 0.16429 | 0.09175 | 0 |  |  |  |
| 10. St Jean le Thomas | 0.29423 | 0.34577 | 0.2428 | 0.31056 | 0.20374 | 0.22491 | 0.23366 | 0.13591 | 0.10694 | 0 |  |  |
| 11. Baie d'Aiguillon | 0.22116 | 0.26427 | 0.1632 | 0.2223 | 0.12154 | 0.14048 | 0.14648 | 0.07662 | 0.04934 | 0.09917 | 0 |  |
| 12. Aiffres | 0.22246 | 0.26882 | 0.16981 | 0.22765 | 0.1248 | 0.14943 | 0.14802 | 0.08139 | 0.04981 | 0.12352 | 0.0073 | 0 |
| 13. Avallon | 0.17648 | 0.23431 | 0.19488 | 0.2422 | 0.1663 | 0.18511 | 0.18763 | 0.17307 | 0.19825 | 0.24087 | 0.17204 | 0.16889 |
| 14. Clérmont-Ferrand | 0.22786 | 0.28201 | 0.22371 | 0.27883 | 0.19509 | 0.19568 | 0.22 | 0.17226 | 0.21989 | 0.27433 | 0.18758 | 0.18271 |
| 15. Alflen | 0.15381 | 0.21051 | 0.19976 | 0.23624 | 0.18154 | 0.18285 | 0.18916 | 0.1879 | 0.20989 | 0.26227 | 0.17952 | 0.18615 |
| 16. Schiltach | 0.19844 | 0.25514 | 0.23039 | 0.26356 | 0.19762 | 0.22338 | 0.22871 | 0.21452 | 0.22448 | 0.26393 | 0.20643 | 0.21262 |
| 17. Loch of Swartmill | 0.4896 | 0.5098 | 0.45562 | 0.50265 | 0.44807 | 0.47583 | 0.48317 | 0.46974 | 0.49919 | 0.58936 | 0.49469 | 0.46317 |
| 18. Ness | 0.54531 | 0.56715 | 0.4982 | 0.5419 | 0.48512 | 0.51291 | 0.5262 | 0.51113 | 0.54284 | 0.64371 | 0.54319 | 0.50685 |
| 19. Whitemill Bay | 0.45585 | 0.52462 | 0.45546 | 0.49327 | 0.40965 | 0.45697 | 0.45384 | 0.46452 | 0.52038 | 0.60522 | 0.51094 | 0.49089 |
| 20. Settiscarth | 0.24013 | 0.27238 | 0.27216 | 0.3138 | 0.24182 | 0.26865 | 0.26118 | 0.28461 | 0.33087 | 0.38845 | 0.30301 | 0.30334 |
| 21. Harray Stenness | 0.2474 | 0.27656 | 0.27482 | 0.30541 | 0.24392 | 0.27151 | 0.26149 | 0.27911 | 0.31522 | 0.37554 | 0.29087 | 0.28707 |
| 22. St Ola | 0.2995 | 0.35354 | 0.32026 | 0.37372 | 0.28968 | 0.33638 | 0.31744 | 0.33457 | 0.37473 | 0.43862 | 0.35641 | 0.34802 |
| 23. Grimness | 0.4906 | 0.53124 | 0.47106 | 0.50829 | 0.46132 | 0.48554 | 0.48878 | 0.48637 | 0.52027 | 0.61662 | 0.51181 | 0.48506 |
| 24. Wind Wick | 0.50524 | 0.54619 | 0.48646 | 0.52655 | 0.47786 | 0.50444 | 0.50739 | 0.50061 | 0.53873 | 0.63623 | 0.52956 | 0.49986 |

|  | 13 | 14 | 15 | 16 | 17 | 18 | 19 | 20 | 21 | 22 | 23 | 24 |
| --- | --- | --- | --- | --- | --- | --- | --- | --- | --- | --- | --- | --- |
| 1. Heerenveen |  |  |  |  |  |  |  |  |  |  |  |  |
| 2. Dinteloord |  |  |  |  |  |  |  |  |  |  |  |  |
| 3. Stalhille |  |  |  |  |  |  |  |  |  |  |  |  |
| 4. Veurne |  |  |  |  |  |  |  |  |  |  |  |  |
| 5. Pihen lès Guînes |  |  |  |  |  |  |  |  |  |  |  |  |
| 6. Fressenneville |  |  |  |  |  |  |  |  |  |  |  |  |
| 7. Daubeuf |  |  |  |  |  |  |  |  |  |  |  |  |
| 8. Thaon |  |  |  |  |  |  |  |  |  |  |  |  |
| 9. Ste Marie du Mont |  |  |  |  |  |  |  |  |  |  |  |  |
| 10. St Jean le Thomas |  |  |  |  |  |  |  |  |  |  |  |  |
| 11. Baie d'Aiguillon |  |  |  |  |  |  |  |  |  |  |  |  |
| 12. Aiffres |  |  |  |  |  |  |  |  |  |  |  |  |
| 13. Avallon | 0 |  |  |  |  |  |  |  |  |  |  |  |
| 14. Clérmont-Ferrand | 0.08026 | 0 |  |  |  |  |  |  |  |  |  |  |
| 15. Alflen | 0.12805 | 0.1556 | 0 |  |  |  |  |  |  |  |  |  |
| 16. Schiltach | 0.15312 | 0.2058 | 0.15912 | 0 |  |  |  |  |  |  |  |  |
| 17. Loch of Swartmill | 0.45637 | 0.49003 | 0.40106 | 0.44707 | 0 |  |  |  |  |  |  |  |
| 18. Ness | 0.49946 | 0.53781 | 0.44083 | 0.49447 | 0.22017 | 0 |  |  |  |  |  |  |
| 19. Whitemill Bay | 0.46842 | 0.49923 | 0.41168 | 0.47713 | 0.61956 | 0.64944 | 0 |  |  |  |  |  |
| 20. Settiscarth | 0.27082 | 0.30924 | 0.21999 | 0.26106 | 0.32017 | 0.39387 | 0.3644 | 0 |  |  |  |  |
| 21. Harray Stenness | 0.26814 | 0.30755 | 0.21004 | 0.25209 | 0.33633 | 0.39611 | 0.38205 | 0.09434 | 0 |  |  |  |
| 22. St Ola | 0.32362 | 0.36137 | 0.26492 | 0.32062 | 0.35892 | 0.39362 | 0.36219 | 0.15451 | 0.13524 | 0 |  |  |
| 23. Grimness | 0.45611 | 0.48492 | 0.40057 | 0.45305 | 0.61973 | 0.647 | 0.63473 | 0.37034 | 0.32862 | 0.40099 | 0 |  |
| 24. Wind Wick | 0.46539 | 0.50527 | 0.41684 | 0.48299 | 0.65066 | 0.68222 | 0.66865 | 0.39264 | 0.34075 | 0.4274 | 0.20479 | 0 |

All *P* < 0.0001 except for Aiffres vs Baie d'Aiguillon: *P* = 0.073


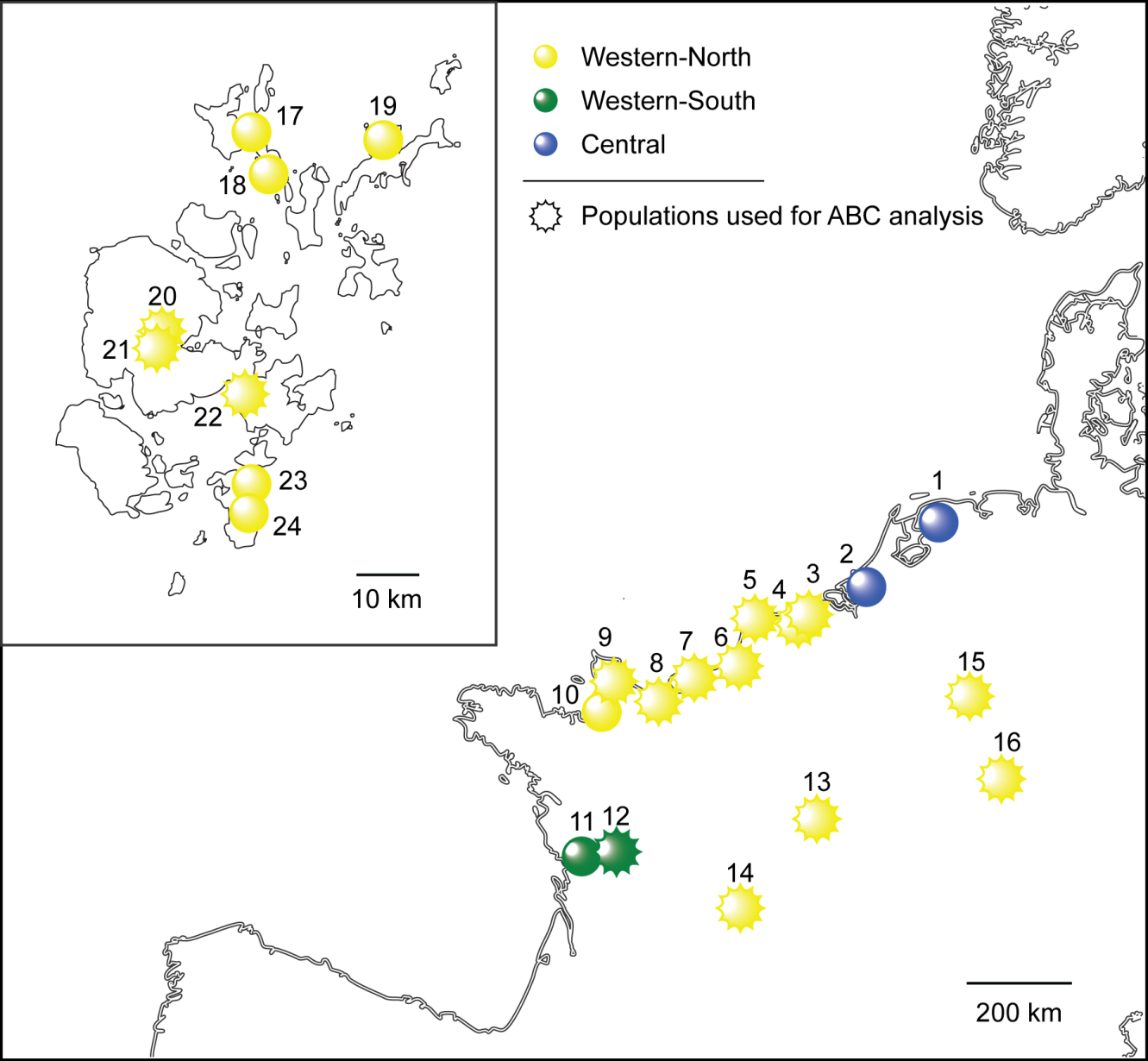


**Fig. S1** Map showing distribution of population samples of modern *M. arvalis* used for microsatellite typing, labelled for mtDNA lineage. Population names as listed in Table 1: 1 – Heerenveen, 2 – Dinteloord, 3 – Stalhille, 4 – Veurne, 5 – Pihen lès Guînes, 6 – Fressenneville, 7 – Daubeuf, 8 – Thaon, 9 – Ste Marie du Mont, 10 – St Jean du Thomas, 11 – Baie d’Aiguillon, 12 – Aiffres, 13 – Avallon, 14 – Clérmont-Ferrand, 15 – Alflen, 16 – Schiltach, 17 – Loch of Swartmill, 18 – Ness, 19 – Whitemill Bay, 20 – Settiscarth, 21 – Harray Stenness, 22 – St Ola, 23 – Grimness, 24 – Wind Wick.


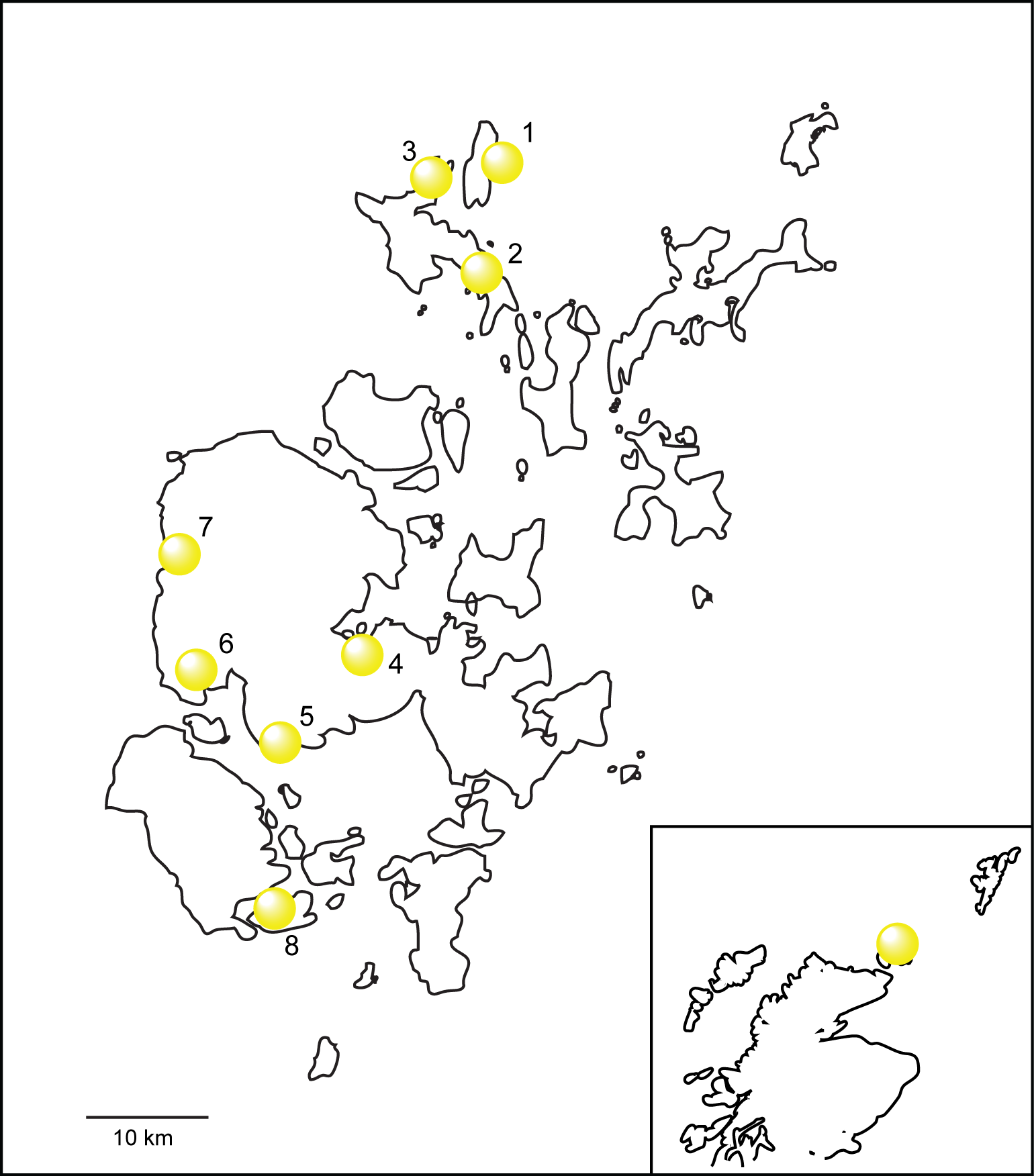


**Fig. S2** Map showing Orkney localities where ancient specimens of *M. arvalis* successfully provided either radiocarbon dates and/or *cytb* sequences. Localities as listed in Tables 2 and S3: 1 - Holm of Papa Westray, Westray, 2 - Point of Cott, Westray, 3 - Pierowall Quarry, Westray, 4 -Quanterness, Mainland, 5 - Earl's Bu, Mainland, 6 - Howe, Mainland, 7 - Skara Brae, Mainland, 8 - Green Hill, South Walls, Hoy.


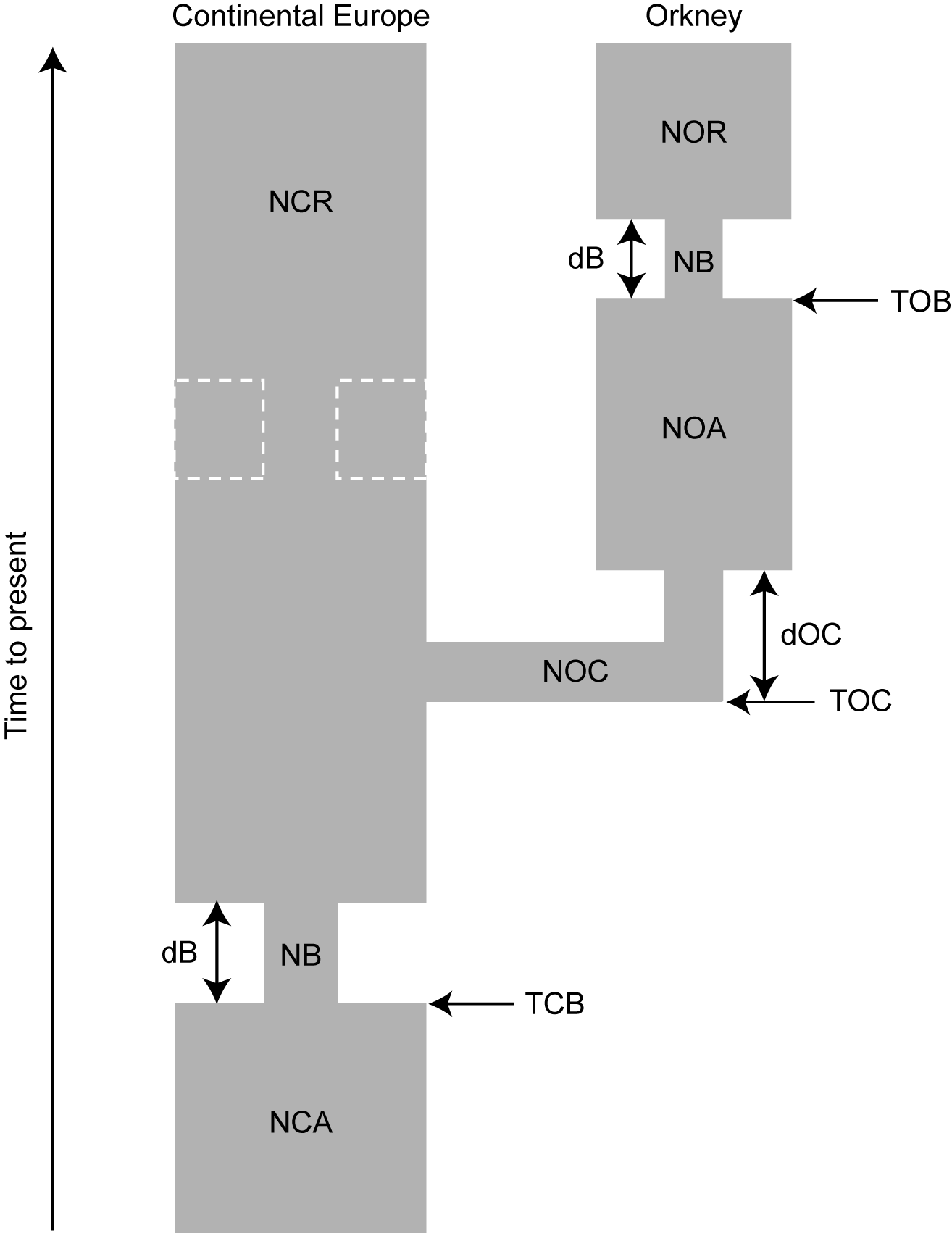


**Fig. S3** Diagram illustrating the ABC model parameters.
